# Maternal metal concentration during gestation and pediatric morbidity in children: an exploratory analysis

**DOI:** 10.1186/s12199-021-00963-z

**Published:** 2021-03-25

**Authors:** Isabella Karakis, Daniella Landau, Roni Gat, Nofar Shemesh, Ofir Tirosh, Maayan Yitshak-Sade, Batia Sarov, Lena Novack

**Affiliations:** 1 Environmental Epidemiology, Ministry of Health, Jerusalem, Israel; 2 Neonatology Department, Soroka University Medical Center, Beer-Sheva, Israel; 3 Clinical Research Center, Soroka University Medical Center, Beer-Sheva, Israel; 4 Faculty of Health Sciences, Ben-Gurion University of the Negev, Beer-Sheva, Israel; 5 Department of Clinical Biochemistry and Pharmacology, Ben-Gurion University of the Negev, Beer-Sheva, Israel; 6 The Fredy and Nadine Herrmann Institute of Earth Sciences, The Hebrew University of Jerusalem, Jerusalem, Israel; 7 Department of Environmental Health, Harvard T.H. Chan School of Public Health, Boston, USA; 8 Department of Public Health, Ben-Gurion University of the Negev, Beer-Sheva, Israel; 9 Negev Environmental Health Research Institute, Soroka University Medical Center, Sderot Rager 151, 84101 Beer-Sheva, Israel

**Keywords:** Heavy metals, Pregnancy, Pediatric, Morbidity, Cohort, Exploratory Analysis

## Abstract

**Background:**

The majority of studies linking exposure to metals with certain health outcomes focus on known toxic metals. Alternatively, this study assesses the extent to which exposure to a wider range of metals during gestation is associated with childhood morbidity.

**Methods:**

We analyzed the concentrations of 25 metals found in urine samples of 111 pregnant women of Arab-Bedouin origin collected prior to birth. In addition, we collected medical records on their offspring for six years following birth, including every interaction with HMOs, local hospitals, and pharmacies.

**Results:**

The main types of morbidities diagnosed and treated during this period were preterm births, malformations, asthma-like morbidity, cardiovascular and behavioral problems, and obesity. Multivariable analysis showed that offspring born before term were more likely to have been exposed to elevated maternal concentrations of zinc, thallium, aluminum, manganese, and uranium, all with adjusted relative risk above 1.40 for an increase by each quintile. Likewise, children with asthma had been exposed to higher levels of magnesium, strontium, and barium at gestation, while behavioral outcomes were associated with elevated biometals, i.e., sodium, magnesium, calcium, selenium, and zinc, as well as higher levels of lithium, cobalt, nickel, strontium, cadmium, vanadium, arsenic, and molybdenum. A heatmap of adjusted relative risk estimates indicates the considerable implications that exposure to metals may have for preterm birth and developmental outcomes.

**Conclusions:**

The current study shows that perinatal exposure to metals is adversely associated with pediatric morbidity. Further such analyses on additional samples are warranted.

**Supplementary Information:**

The online version contains supplementary material available at 10.1186/s12199-021-00963-z.

## Background

Exposure to heavy metals, the majority of which are highly toxic, can result in severe human morbidities. Lead (Pb), arsenic (As), and mercury (Hg) head the list, followed by aluminum (Al), cadmium (Cd), chromium (Cr), nickel (Ni), titanium (Ti), and others, whose presence is not essential to the human body. As a result, exposure to high concentrations is likely to be adversely associated with health outcomes. At the same time, other metals, including calcium (Ca), cobalt (Co), copper (Cu), iron (Fe), magnesium (Mg), manganese (Mn), molybdenum (Mo), selenium (Se), sodium (Na), and zinc (Zn), are considered essential for human physiology [1]. There is no consensus on the list of essential metals in medical literature; however, some researchers limit them to only Na, potassium (K), Mg, Ca, Zn, Se, and Cu [2, 3]. This group, also known as “biometals,” is characterized by a U-shaped association with health outcomes, whereas no U shape has been reported for non-essential metals [1].

Since toxic metals are known to cross the placenta and blood-brain barrier and deposit in fetal tissues, children are likely to be susceptible to metal exposure early, starting from the time of gestation. For this reason, maternal metal concentration is frequently used as a proxy for that of a fetus when studying health outcomes.

Maternal elevated concentrations of Al, As, Cd, Hg, Ni, and Mn have been reported in relation to newborn *congenital defects* at birth [4–7]. *Small-for-gestational age* (SGA) status at birth has been adversely linked to Cd [8] and As [8, 9]. Likewise, *preterm birth* was reported associated with Cd [10, 11] and Pb [12]. Nevertheless, the association between Pb, Cd, As, Cr, or Hg and preterm birth was not always confirmed [13, 14].

A recent review by Allen provided an evidence of an association between prenatal Pb exposure and *neurological* and *cognitive impairment* [15]. The findings of several other studies support this link for Pb [16, 17], as well as for Mn [18, 19], Hg [19], As [19], and Cd [20, 21]. However, some contradictory findings have also been reported for Cd [19, 22–24]. The lack of association in those cases might have been a result of adjustment to smoking, known to be related to Cd levels [25] as well as to cognitive function [26], and therefore a potential confounder in the association between the two.

*Pulmonary* function indices and asthma in children were linked with Pb [27, 28], Mn, Ni, and Cr [29, 30], as well as Co and Zn [31]. Likewise, maternal Pb has been reported in association with *elevated blood pressure* in 5-year-old children [32] and prenatal exposure to Cd—with an increased risk of *obesity* at the age of five [33].

The Bedouin-Arab population of southern Israel is known for elevated rates of childhood morbidity [34] usually related to its low socio-economic status, previous and to some degree still prevalent semi-nomadic life style, temporary housing conditions, high rates of smoking among males, and consanguineous marriages [34, 35].

Because of the vulnerability of the Bedouin-Arab community, the extensive monitoring of hazardous exposures, in an attempt to identify and eliminate modifiable factors related to morbidity, is required. In the current analysis, we aim to explore the possible links between maternal exposure to a wide range of heavy metals at gestation with birth outcomes and the health conditions of 5 to 6-year-old offspring.

## Methods

### Study population

We investigated the subset of a cohort of mothers and their newborns of Bedouin-Arab origin enrolled in our study between Dec 2011 and Mar 2013. Enrollment procedures and characteristics of the study population are described in a previous study [36]. Briefly, all women of Bedouin-Arab origin arriving at the obstetrics emergency department for a delivery at Soroka University Medical Center (SUMC) during the regular working hours were approached by an Arabic-speaking interviewer and invited to participate in the study. Upon signing the informed consent form, the women’s spot urine sample was collected, although not all women could comply with this requirement given their condition and a relatively short time to the onset of delivery. The questionnaire on the demographical and clinical characteristics of the women, as well as a description of possible hazardous factors in their immediate household, was administered during the hospitalization following the delivery. In all, the cohort included 1823 women.

In order to be included in the current analysis, the women had to be the members of “Clalit” health maintenance organization (HMO), 18 years and older, and to have an available urine sample collected prior to birth and the newborns had to survive their birth hospitalization. The membership in Clalit HMO allowed the researchers an access to the subjects’ medical records besides the information in the hospital system. This additional information was especially important for retrieval of pediatric diagnoses for morbidities not requiring hospitalization. Since members of the Clalit HMO represent close to 70% of the local population, this restriction is not expected to affect the generalizability of the study conclusions. In all, 1437 of the initial cohort had Clalit HMO membership.

The study hospital (SUMC) is located in Beer-Sheva, a metropolis city in the Negev desert, in southern Israel. This is the only hospital providing tertiary and outpatient services in the Negev region to a population of 1 million residents (see supplementary material, Figure 1, for the area map along with the subjects’ residence locations).

### Exposure to metals

We estimated the concentrations of 25 metals in urine samples collected prior to delivery. Samples were stored at – 20 °C prior to their testing 5 to 6 years later. An Agilent 7500 Series inductively coupled plasma mass spectrometer (ICP-MS) (Agilent Technologies, Tokyo, Japan) was used to assess the metal content of each sample. The list of metals included Na, K, Mg, Ca, Zn, Se, Cu, Li, Co, Ni, Tl, Al, Cr, strontium (Sr), Cd, barium (Ba), beryllium (Be), vanadium (V), Fe, As, molybdenium (Mo), Pb, silver (Ag), Mn, and uranium (U). Testing was performed in a trace metal clean room, a laboratory in the Institute of Earth Sciences at the Hebrew University of Jerusalem. Values that fell below the level of quantification (LOQ) were imputed by LOQ = 0.01 divided by the square root of two [37]. A more detailed description of the lab methodology has been reported elsewhere [38]. No adjustment was performed for creatinine, as these levels were deemed not to vary significantly within the group of all relatively young female subjects.

### Health outcomes

We obtained access to all medical records prepared by local hospital and/or HMO personnel. These hospitalization records relied on the admission-transfer-discharge (ATD) database and included details concerning children’s complications and diagnoses during birth, subsequent hospitalizations, and emergency room and outpatient clinic visits. HMO records included visits to primary physicians, specialists’ clinics, and records on all medications purchased for a child through an HMO pharmacy. It should be noted that pharmacies in Israel sell all the chronic and the majority of over-the-counter (OTC) medications, both with a substantial discount when bought with an HMO membership. For that reason, the HMO pharmacy records are expected to almost fully cover the medication purchases. The data platforms listed above represent the comprehensive list of medical records collected for each child in the analysis. Based on these sources, we were able to retrieve the complete information on diagnoses, medications, and types of clinics visited by children during the follow-up.

The following categories of morbidities were verified: preterm delivery and small-for-gestational age (SGA), malformations, asthma and cardiovascular morbidity, behavioral/developmental disorders, obesity, and malignancies.

We defined *preterm delivery* as delivery at a gestational age equal to or less than 37.0 weeks [39, 40]. Of note, in the majority of deliveries, gestational age is rounded up to the whole number of weeks; therefore, gestational age “37.0” frequently included deliveries taking place a few days before 37.0. Small-for-gestational age (SGA), defined by a diagnosis assigned to newborns at delivery, refers to newborns under the 10th percentile in weight for their gestational age [41, 42]. *Malformations*’ diagnosis in an International Classification of Diseases 9th revision (ICD-9) system is based on the neonatologist’s evaluation shortly following delivery together with diagnoses received throughout a child’s encounters with the medical system, as recorded in the ATD and HMO databases (Table S2, supplementary material). Malformations’ diagnoses are further classified by body systems as well as by their severity, i.e., minor or major.

*Asthma-like* morbidity refers to any encounters with the medical system when an ICD-9 diagnosis related to asthma was recorded or an asthma medication was purchased. The same approach was used for *malignancies*, *cardiovascular*, *behavioral* and *developmental morbidities*, and *obesity*. The specific codes used in the study are presented in Table S2 (supplementary material).

### Statistical analysis

Metal concentrations in urine were reported as a geometric means along with a 95% confidence interval (CI) and compared by subjects’ morbidity status using a ratio *t* test in a univariate analysis (crude comparisons are not shown). Concentrations were ranked into quintiles, and their associations with health outcomes were explored visually.

We adjusted for individual risk factors using a Poisson regression model with a health outcome as a dependent variable and metal concentration in quintiles and other factors as independent parameters. We used the same list of covariates for models exploring associations with all health outcomes, and they included maternal age, parity, newborn gender, and preterm birth. Covariates in the model of preterm birth included only maternal age, parity, and newborn gender. The detailed information on the models is presented in Table S3 (supplementary material).

The point estimates of an association were expressed as relative risk (RR). In order to ensure the robustness of the model inference, we conservatively chose the sandwich estimator approach for standard error calculation. Adjusted point estimates of RR are presented in a heatmap reflecting a potential range of an effect of metals on health outcomes.

In view of the explorative nature of this analysis, performed on SAS 9.4 and R software, and a relatively small sample size, we did not correct for multiple comparisons. The code for the main analysis is provided (supplementary materials).

## Results

In all, 111 of women and their 111 newborns had a stored maternal urine sample available for chemical analysis. There were no twins in the sub-sample. Mothers were on average (mean ± SD) 28.1 ± 6.3 years old, with more than half of them giving birth to their fourth child (Table 1). Most newborns were born at term at an average weight of 3287.3 ± 457.5 g; 10.8% were born at 37.0 weeks of gestation or earlier. The study follow-up fell within a range of 5.4-6.9 years, with  more than half of the children underwent above  6.1 years of follow-up. Women and their offspring were not different from the main cohort, as shown in its comparison to 1437/1823 women initially enrolled in the study and limited to Clalit HMO members (Table S1, supplementary materials).

**Table 1:** Demographic characteristics and clinical outcomes in the study population

Subjects’ characteristics	Study population (*N* = 111 newborns) (*N* = 111 deliveries)
*Demography*	
Maternal age, years	
Mean ± SD (*n*)	28.1 ± 6.3 (110)
Median	26.9
Min; max	18.4; 41.7
Parity, % (*n*/*N*)	
1	27.3 (30/110)
2–5	42.7 (47/110)
6+	30.0 (33/110)
Gestational age, weeks	
Mean ± SD (*n*)	39.4 ± 1.8 (111)
Median	39.0
Min; max	32.0; 44.0
Infant weight, grams	
Mean ± SD (*n*)	3287.3 ± 457.5 (111)
Median	3345.0
Min; max	1725.0; 4165.0
Small-to-gestational age (SGA), % (*n*/*N*)	6.4 (7/109)
Infant male gender, % (*n*/*N*)	54.1 (60/111)
Children’s age at follow-up, years	
Mean ± SD (*n*)	6.1 ± 0.3 (111)
Median	6.1
Min; max	5.4; 6.9
*Clinical outcomes,*	
Preterm delivery, % (*n*/*N*)	10.8 (12/111)
Malformations diagnosed at birth or follow-up, % (*n*/*N*)	7.2 (8/111)
Malformations’ severity, % (*n*/*N*)	
Major	1.5 (5/111)
Minor	2.7 (3/111)
Asthma morbidity, % (*n*/*N*)	
Reported as treated	69.4 (77/111)
Diagnosed	51.4 (57/111)
Cardiovascular morbidity, % (*n*/*N*)	
Reported as treated	10.8 (12/111)
Diagnosed	0 (0/111)
Behavioral conditions^a^, % (*n*/*N*)	
Reported as treated	6.3 (7/111)
Diagnosed	0.9 (1/111)
Obesity, % (*n*/*N*)	
Reported as treated	5.4 (6/111)
Diagnosed	5.4 (6/111)
Burden of disease, % (*n*/*N*)	
0	26.1 (29/111)
1–2	69.4 (77/111)
3–4	3.6 (4/111)
5–6	0.9 (1/111)

^a^Patients who visited a developmental outpatient clinic at the ages 0.5–3 years (in all 6 patients) and/or diagnosed with a behavioral disorder (1 patient—autism and developmental delay).

Table 1 shows the list of health outcomes deemed related to environmental exposures, according to the literature. For more than half of the children, an asthma-like diagnosis was recorded in their medical charts at least once at a median age of 0.6 years, while 69.4% of all children were treated at least once for asthma-related symptoms in an emergency room or by a medication. In addition, approximately 10.8% of children at a median age of 0.8 years were referred to specialists for cardiovascular-related morbidities, although none were diagnosed with a cardiovascular disease. Over 6% of children were treated for developmental or behavioral disorders, the earliest treatment given to a 0.3-year-old child and the latest to a 6-year-old. Overall, six were referred to an outpatient developmental clinic where one child was diagnosed with developmental delay and autism. Obesity was diagnosed in 5.4% of the sample, usually around the age of 5 years or later. There were no malignancies among the 111 children in the sample, and no mortality cases were recorded. Similar to its baseline characteristics, the population of the sub-sample did not vary from its main cohort in any of the outcomes (Table S1, supplementary material).

Table 2 shows the metal concentrations in the study population. Although, based on a small sample, their distribution does not vary from the findings reported by the authors elsewhere, alongside comparison of the obtained concentrations to the values reported in NHANES population [38]. However, the comparison to a sample of 31 women in Sweden [43] and a sample of 968 women in France [44] indicated excessive levels of certain metals, e.g., Se, Al, Co, Cr, and Pb (Table 2).

**Table 2:** Internal dose of metals in the study population

	Metal (*n* = 111)	Geometric mean (GM)	95% CI	Min; max	Percentiles				Comparison with similar populations	
					20th	40th	60th	80th	GM (90th)^a^ 21–64-year-old females (*n* = 31)	GM (95% CI)^b^ 20–60-year-old females (*n* = 968)
Biometals	Ca, ppm	19.68	12.28; 31.53	0.01; 287.41	10.11	27.13	50.82	93.10		
	Cu, ppb	12.10	8.55; 17.12	0.01; 2861.69	8.38	12.54	18.33	31.74	5.06 (7.25)	
	K, ppm	998.88	604.84;649.63	0.01; 5633.91	904.05	1556.99	2070.66	3228.45		
	Mg, ppm	15.17	9.41; 24.43	0.01; 160.22	10.92	23.84	40.28	62.66		
	Na, ppm	1629.31	969.30; 2738.74	0.01; 10832.98	1704.94	2538.68	3615.22	4824.97		
	Se, ppb	19.58	13.73; 27.91	0.01; 226.59	16.54	22.97	33.18	47.78	13.1 (18.3)	
	Zn, ppb	146.51	93.61; 229.32	0.01; 4862.63	86.84	173.24	301.37	487.03	172 (316)	265 (251–280)
Non-essential metals	Ag, ppb	0.04	0.03; 0.08	0.01; 17.13	0.01	0.01	0.01	7.29		
	Al, ppb	6.14	3.80; 9.90	0.01; 97.27	4.34	11.27	17.17	26.52		2.28 (2.07–2.51)
	As, ppb	3.59	2.33; 5.52	0.01; 146.21	1.48	4.41	9.17	16.60	32.2 (123)	18.2 (17.0–19.4)
	Ba, ppb	1.29	0.89; 1.87	0.01; 337.47	0.64	1.32	2.49	4.40		
	Be, ppb	0.11	0.07; 0.16	0.01; 2.63	0.01	0.11	0.30	0.72		0.004 (0.004–0.005)
	Cd, ppb	0.20	0.16; 0.26	0.01; 2.75	0.10	0.20	0.30	0.50	0.11 (0.26)	0.39 (0.37–0.42)
	Co, ppb	0.93	0.73; 1.19	0.01; 4.37	0.46	0.92	1.40	2.47	0.31 (0.69)	0.66(0.63–0.69)
	Cr, ppb	0.63	0.44; 0.90	0.01; 10.09	0.28	0.71	1.18	2.19	0.064 (0.10)	0.39 (0.36–0.42)
	Fe, ppb	1.17	0.54; 2.53	0.01; 1640.82	0.01	3.33	16.54	39.43	2.34 (5.14)	
	Li, ppb	9.64	5.10; 9.52	0.01; 74.99	5.18	8.15	11.20	16.21	9.22 (16.1)	
	Mn, ppb	0.03	0.02; 0.04	0.01; 4.81	0.01	0.01	0.01	0.39	0.10 (0.25)	0.29 (0.27–0.31)
	Mo, ppb	7.23	3.86; 13.55	0.01; 604.57	4.53	19.64	34.76	64.20	31.3 (63.4)	
	Ni, ppb	1.23	0.95; 1.60	0.01; 8.31	0.65	1.28	2.20	3.02	0.91 (1.57)	2.10 (1.98–2.21)
	Pb, ppb	0.17	0.09; 0.32	0.01; 62.02	0.01	0.01	0.43	9.66	0.28 (0.43)	0.90 (0.84–0.95)
	Sr, ppb	72.73	48.25; 109.65	0.01; 556.97	45.41	88.50	135.57	249.20		
	Tl, ppb	0.04	0.03; 0.05	0.01; 0.62	0.01	0.01	0.11	0.20		0.20 (0.19–0.21)
	V, ppb	0.04	0.03; 0.06	0.01; 1.22	0.01	0.01	0.10	0.28	0.011 (0.018)	0.24 (0.22–0.26)
	U, ppb	0.01	0.01; 0.01	0.01; 0.11	0.01	0.01	0.01	0.01	1.67 (2.82)	

^a^Swedish population

^b^French population

Metals’ concentrations by the outcomes at study are graphically described in Figures 2 a–f (supplementary material).

**Fig. 1:**
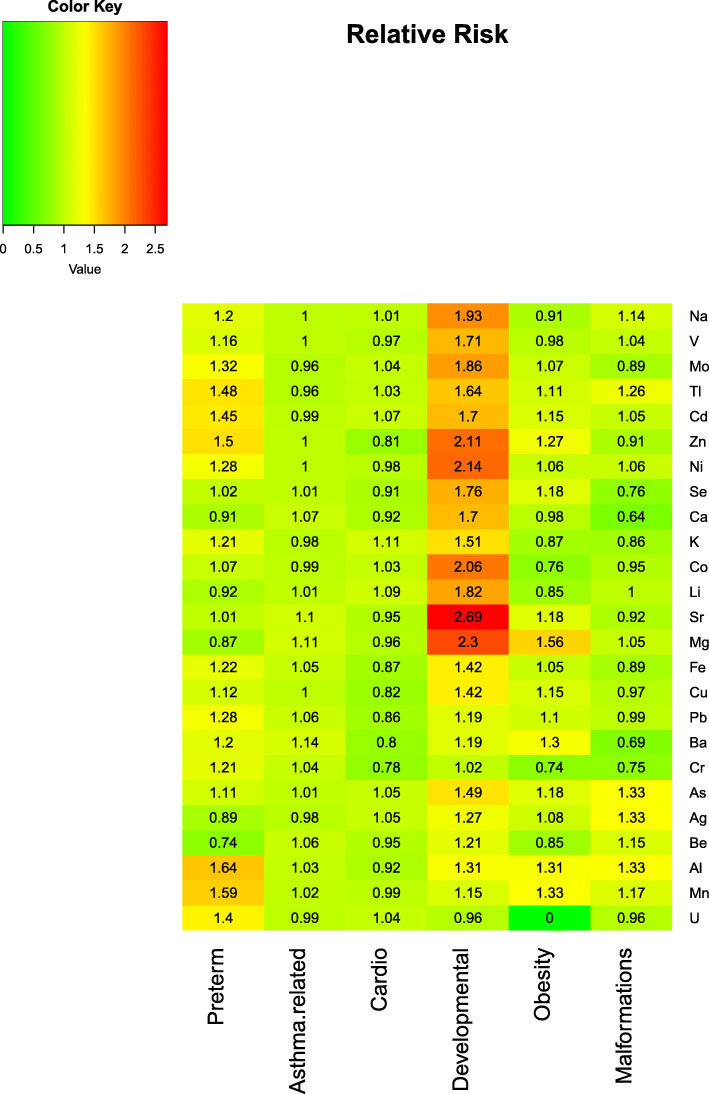
Heatmap of association between internal dose of metals in quintiles and presence of a clinical outcome at birth, adjusted to maternal and newborn clinical background and expressed as relative risk (the color scale ranges from green assigned to protective factors to red assigned to risk factors with relative risk > 1)

Women who delivered preterm were more likely to have elevated concentrations of Zn, Tl, Al, Mn, and U with an RR of 1.40–1.64 times higher compared to in term deliveries (see Table 3 and Fig. 1 showing a heatmap of the associations presented in Table 3). Cd, Ni, and Pb were associated with preterm delivery, although with a borderline significance (0.05 < *p* value < 0.1) and with comparable point estimates. Asthma was linked to high concentrations of Mg, Sr, and Ba, all featured by a relatively small effect of RR close to 1.1. The behavioral and developmental group of disorders was the one most frequently associated with elevated metals, with the majority of them biometals, as well as Li, Co, Ni, Sr, Cd, V, As, and Mo. All point estimates were greater than PR = 1.42 and were different in their majority from the metals linked to preterm birth in this study.

**Table 3:** Association between internal dose of metals in quintiles and presence of a clinical outcome at birth, adjusted to maternal and newborn clinical background and SGA^1^,^2^

	Metal (in quintiles)	Preterm delivery (*n* = 12) vs none (*n* = 97)	Asthma (*n* = 77) vs none (*n* = 34)	Cardiovascular (*n* = 12) vs none (*n* = 99)	Behavioral or developmental (*n* = 7) vs none (*n* = 104)	Obesity (*n* = 6) vs none (*n* = 105)	Malformations (*n* = 8) vs none (*n* = 103)
Biometals	Ca, ppm	0.91 (0.626)	1.07 (0.151)	0.92 (0.687)	1.70 (0.026)	0.98 (0.946)	0.64 (0.121)
	Cu, ppb	1.12 (0.487)	1.00 (na)	0.82 (0.371)	1.48 (0.080)	1.15 (0.679)	0.97 (0.909)
	K, ppm	1.21 (0.198)	0.98 (0.637)	1.11 (0.640)	1.51 (0.095)	0.87 (0.634)	0.86 (0.599)
	Mg, ppm	0.87 (0.409)	1.11 (0.027)	0.96 (0.847)	2.30 (0.018)	1.56 (0.239)	1.05 (0.771)
	Na, ppm	1.20 (0.204)	1.00 (0.999)	1.01 (0.969)	1.93 (0.016)	0.91 (0.740)	1.14 (0.584)
	Se, ppb	1.02 (0.881)	1.01 (0.792)	0.91 (0.653)	1.76 (0.025)	1.18 (0.570)	0.76 (0.379)
	Zn, ppb	1.50 (0.033)	1.00 (0.997)	0.81 (0.357)	2.11 (0.022)	1.27 (0.483)	0.91 (0.751)
Non-essential metals	Ag, ppb	0.89 (0.500)	0.98 (0.534)	1.05 (0.771)	1.27 (0.273)	1.27 (0.273)	1.33 (0.094)
	Al, ppb	1.64 (0.003)	1.03 (0.536)	0.92 (0.670)	1.31 (0.331)	1.31 (0.510)	1.33 (0.223)
	As, ppb	1.11 (0.516)	1.01 (0.794)	1.05 (0.820)	1.49 (0.041)	1.18 (0.614)	1.33 (0.344)
	Ba, ppb	1.20 (0.300)	1.14 (0.004)	0.80 (0.262)	1.19 (0.503)	1.30 (0.565)	0.69 (0.191)
	Be, ppb	0.74 (0.091)	1.06 (0.209)	0.95 (0.775)	1.21 (0.270)	0.85 (0.653)	1.15 (0.579)
	Cd, ppb	1.45 (0.084)	0.99 (0.874)	1.07 (0.760)	1.70 (< 0.001)	1.15 (0.702)	1.05 (0.856)
	Co, ppb	1.07 (0.638)	0.99 (0.892)	1.03 (0.888)	2.06 (0.003)	0.76 (0.208)	0.95 (0.830)
	Cr, ppb	1.21 (0.302)	1.04 (0.323)	0.78 (0.171)	1.02 (0.938)	0.74 (0.379)	0.75 (0.239)
	Fe, ppb	1.22 (0.202)	1.05 (0.238)	0.87 (0.511)	1.42 (0.080)	1.05 (0.936)	0.89 (0.623)
	Li, ppb	0.92 (0.559)	1.01 (0.853)	1.09 (0.672)	1.82 (0.010)	0.85 (0.467)	1.00 (0.998)
	Mn, ppb	1.59 (< 0.001)	1.02 (0.585)	0.99 (0.942)	1.15 (0.441)	1.33 (0.258)	1.17 (0.331)
	Mo, ppb	1.32 (0.129)	0.96 (0.315)	1.04 (0.856)	1.86 (0.016)	1.07 (0.796)	0.89 (0.655)
	Ni, ppb	1.28 (0.090)	1.00 (na)	0.98 (0.942)	2.14 (0.014)	1.06 (0.837)	1.06 (0.809)
	Pb, ppb	1.28 (0.087)	1.06 (0.147)	0.86 (0.387)	1.19 (0.315)	1.19 (0.315)	0.99 (0.953)
	Sr, ppb	1.01 (0.972)	1.10 (0.029)	0.95 (0.813)	2.69 (0.009)	1.18 (0.553)	0.92 (0.758)
	Tl, ppb	1.48 (0.013)	0.96 (0.394)	1.03 (0.874)	1.64 (0.191)	1.11 (0.718)	1.26 (0.390)
	V, ppb	1.16 (0.292)	1.00 (na)	0.97 (0.842)	1.71 (0.017)	0.98 (0.961)	1.04 (0.826)
	U, ppb	1.40 (0.009)	0.99 (0.821)	1.04 (0.808)	0.96 (0.839)	0.96 (0.839)	0.96 (0.849)
Covariates in the model		Maternal age, parity, newborn gender	Maternal age, parity, newborn gender, preterm birth				

^1^The table presents a relative risk (RR) reflecting a risk of a health outcome as a function of metals concentrations

^2^na - not available based on the current analysis

Upon closer examination of five children with a high burden of disease (3 morbidities, etc.; see Table 1), higher concentrations were recorded for most of the metals, especially Ni and As. We did not identify any spatial clusters with respect to morbidity type or elevated metal concentrations.

Finally, as part of a sensitivity analysis, we investigated the possibility of a U-shape association linked to biometals and morbidities. For this purpose, we defined the exposure to a metal as a concentration in the lowest and highest quintiles (data not shown). This U-shape association was supported only in the case of Ca and specifically in its association with behavioral and developmental morbidities, with RR = 5.62 (*p* value = 0.022) for the lowest or highest quintiles of Ca.

Cardiovascular outcomes and obesity were not associated with the metals, independent of other risk factors. Malformations were associated with Ag with PR = 1.33, but at a borderline significance (*p* value = 0.094).

## Discussion

We aimed to describe a possible link between maternal exposure to metals during gestation and pediatric morbidity in children aged 0 to 6 years. The vast majority of similar investigations done to this day were limited in the number of either metals tested for the main biomarker of exposure or pediatric morbidity [45, 46]. In contrast, 25 metals and all clinical conditions developed by children were at the investigators’ disposal in the current analysis.

The study population was characterized by relatively high rates of asthma-like illness, with more than half of the children diagnosed and approximately 70% of these treated for asthma. These high rates of disease are consistent with another local study in which researchers reported comparable numbers for asthma-like symptoms in the Bedouin population [47]. Moreover, the rate of malformations was high, amounting to 9.5% in the study cohort and 7.2% in the sub-sample. It should be noted that these numbers might underestimate the actual rate, since the newborns excluded from the current analysis due to antepartum or peripartum mortality were most probably those with major malformations [4, 34].

We recorded multiple links between morbidities and perinatal exposure to metals in both univariate and adjusted analyses. For instance, preterm delivery could be attributed to perinatal exposure to metals such as Tl, Al, and U as well as Cd and Pb—at significance level < 0.1. Studies of these two metals have shown contradictory results; in other words, while some studies support the link for Cd [10, 11] and for Pb [12], others do not confirm these findings [13, 14]. This inconsistency may be related to different testing matrices like a spot urine, as in case of our study, or serum [14]. These two elements are also closely related to smoking, a potential confounder of associations found. In the current study, any adjustments would not be effective, as children were exposed to second-hand smoke (from their fathers) in more than 92% of cases (data not shown).

Of note, metals linked with preterm delivery in this study were different from metals linked with behavioral and developmental outcomes, indicating some degree of specificity of exposure in its association with an outcome it may potentially produce.

Our analysis of behavioral outcomes showed a link to a slightly different set of metals, including Li, Co, Ni, Sr, V, As, Mo, and Cd. We found no previous studies that explored the association of these metals with behavioral morbidity; most focus on Cd and Pb [15–17, 20, 21]. These together with the metals linked to preterm delivery may all represent industry-related exposure. An additional exploration of morbidity cases did not reveal any residential clusters, although our sub-sample was too small for any meaningful epidemiological investigation of an exposure source.

Despite the fact that an asthma-like illness outcome was not associated with exposure to metals with the exception of Mg, Sr, and Ba, this outcome occurred so commonly as to produce a valid analysis specific to the cause. Malformations were found to be related only to Ag, but at a borderline significance (*p* value < 0.1) when adjusted for other factors. The current study adds to the findings of a relatively short list of studies on the association of metal with birth defects: one shows an adverse effect of As, Pb, Cd, and Cu on neural tube defects in infants [48], another adversely links exposure to Li use in pregnancy with cardiac malformations [49], and a third, focusing on a different sub-sample of the current cohort, shows the adverse association of Al with minor malformations at birth [4]. As previously stated, the malformations we observed do not represent the complete picture of this morbidity; therefore, the current analysis is likely biased toward less severe cases.

Our findings on non-essential metals partially confirm existing theories on their U-shaped impact, i.e., whereas an element should be present within its normal range, and levels too low or too high may present a risk factor for health [1]. We recorded this type of association only for Ca and developmental outcomes, but not for other elements. At the same time, cardiovascular outcome was only negatively associated with essential metals, i.e., subjects with a cardiovascular outcome were more likely to have low levels of Zn. However, this finding was not confirmed in a multivariable analysis.

Our sample was almost identical to that of the study cohort in terms of baseline characteristics and morbidity rates. This similarity confirms the appropriateness of the subgroup’s random selection, as well as enables the extrapolation of our conclusions to a larger population.

The current analysis has few *limitations*, the most obvious being its relatively small size and non-conservative approach which precludes correction of significance levels to the investigation of multiple metals and outcomes; this approach corresponds to the explorative nature of our analysis and promoted some hypothesis-generating findings worthy of further investigation.

A further limitation concerns possible misclassification based on our definitions of morbidity, where an outcome is defined by a diagnosis recorded at any point in life or treatment ever administered if deemed related to that disease. This is a frequent pitfall of definitions based on medical records, which although tending to attenuate measured associations toward the null hypothesis are not expected to bias study conclusions.

Lastly, we investigated a sample of Bedouin-Arab women and their offspring who differ in levels of hazardous exposures from the neighboring Jewish population, yet are treated by the same health services. Nevertheless, links between objectively measured metal concentrations and morbidity based on medical records rather than on self-reporting, adjusted to main risk factors, should not be limited to this ethnic group but should also hold true for other populations.

*To conclude*, the current study offers an analysis of a large number (25 in all) of metals that to date have not been explored for their relation to pediatric morbidity. Behavioral and developmental disorders and preterm delivery were associated with heavy metals detected in maternal urine at pregnancy, independent of other factors.

Our analysis provides many hypotheses-generating findings that warrant further exploration on a larger sample.

## Supplementary Information

**Additional file 1: Table S1**: Demographic characteristics and clinical outcomes in the study population and the initial cohort, Clalit HMO members

**Additional file 2: Table S2**: ICD-9 codes used for health outcome definitions

**Additional file 3: Table S3**: Association between maternal and newborn clinical background and presence of a clinical outcome at birth

**Additional file 4: Figure S1**: Study area map and geographical distribution of the study population. Figure legend: the blue dots in the map represent the subjects' residence location. (JPEG 45 kb)

**Additional file 5: Figure S2**: Graphical presentation of the metal concentrations by outcomes

**Additional file 6.**

## Data Availability

Data supporting the findings will be provided upon request and only following an approval by the local IRB committee.

## Abbreviations

Ag: Silver
Al: Aluminum
As: Arsenic
Ba: Barium
Be: Beryllium
Ca: Calcium
Cd: Cadmium
Cr: Chromium
Co: Cobalt
Cu: Copper
Fe: Iron
Hg: Mercury
K: Potassium
Mg: Magnesium
Mn: Manganese
Mo: Molybdenum
Na: Sodium
Ni: Nickel
Pb: Lead
Se: Selenium
Sr: Strontium
Ti: Titanium
U: Uranium
V: Vanadium
Zn: Zinc
ATD: Admission-transfer-discharge
CI: Confidence interval
HMO: Health maintenance organization
ICD-9: International Classification of Diseases 9th revision
ICP-MS: Inductively coupled plasma mass spectrometer
LOQ: Level of quantification
NHANES: National Health and Nutrition Examination Survey
OTC: Over-the-counter
RR: Relative risk
SGA: Small-for-gestational age
SUMC: Soroka University Medical Center
