# Supplementary material for: Maternal metal concentration during gestation and pediatric morbidity in children: an exploratory analysis
Source: Environ Health Prev Med. 2021 Mar 25;26:40. doi: 10.1186/s12199-021-00963-z (PMC7995788; doi:10.1186/s12199-021-00963-z)
Supplement: Supplementary file 1 — Additional file 1: Table S1: Demographic characteristics and clinical outcomes in the study population and the initial cohort, Clalit HMO members [file 12199_2021_963_MOESM1_ESM.docx]

Table 1: Demographic characteristics and pediatric outcomes in the study population and its sub-set with metals' concentration in urine, Clalit HMO members

| *Demography* | Study cohort  (N=1441 newborns) (N=1437 deliveries) | Sub-sample  (N=111 newborns)  (N=111 deliveries) |
| --- | --- | --- |
| Maternal age, years  Mean±SD (n)  Median  Min; Max | 27.6±6.0 (1434)  26.5  18.0; 46.3 | 28.1±6.3 (110)  26.9  18.4; 41.7 |
| Parity  1  2-5  6+ | 26.8 (384/1434)  49.9 (716/1434)  23.3 (334/1434) | 27.3 (30/110)  42.7 (47/110)  30.0 (33/110) |
| Gestational Age, weeks  Mean±SD (n)  Median  Min; Max | 39.2±2.0 (1437)  39.4  25.0; 44.0 | 39.4±1.8 (111)  39.0  32.0; 44.0 |
| Preterm delivery (≤37.0 weeks) | 13.5 (193/1435) | 10.8 (12/111) |
| Infant Weight, grams  Mean±SD (n)  Median  Min; Max | 3194.3±537.1 (1437)  3235.0  770.0; 5140.0 | 3287.3±457.5 (111)  3345.0  1725.0; 4165.0 |
| Small-to-Gestational Age (SGA), % (n/N)  Large-to-Gestational Age (LGA), % (n/N) | 4.0 (56/1408)  6.5 (92/1408) | 6.4 (7/109)  0 (0/109) |
| Infant male gender, % (n/N) | 53.3 (766/1438) | 54.1 (60/111) |
| Children's age at follow-up, years  Mean±SD (n)  Median  Min; Max | 6.1±0.3 (1437)  6.0  5.4; 6.9 | 6.1±0.3 (111)  6.1  5.4; 6.9 |
| *Pediatric outcomes* |  |  |
| Preterm delivery, % (n/N) | 12.8 (184/1438) | 10.8 (12/111) |
| Asthma-like morbidity | 74.3 (1071/1441) | 69.4 (77/111) |
| Cardiovascular morbidity | 8.8 (127/1441) | 10.8 (12/111) |
| Behavioral and developmental morbidity | 6.5 (93/1441) | 6.3 (7/111) |
| Obesity, % (n/N) | 5.8 (83/1441) | 5.4 (6/111) |
| Malformations, % (n/N) | 9.6 (138/1441) | 7.2 (8/111) |
