# Supplementary material for: Maternal metal concentration during gestation and pediatric morbidity in children: an exploratory analysis
Source: Environ Health Prev Med. 2021 Mar 25;26:40. doi: 10.1186/s12199-021-00963-z (PMC7995788; doi:10.1186/s12199-021-00963-z)
Supplement: Supplementary file 2 — Additional file 2: Table S2: ICD-9 codes used for health outcome definitions [file 12199_2021_963_MOESM2_ESM.docx]

**Table 2**: ICD-9 codes used for health outcome definitions^1^

| **Diagnosis** | **Codes** |
| --- | --- |
| *Malformations* | |
| Anencephalus and similar anomalies | 740 |
| Spina bifida | 741 |
| Other congenital anomalies of nervous system | 742 |
| Congenital anomalies of eye | 743 |
| Congenital anomalies of ear, face, and neck | 744 |
| Bulbus cordis anomalies and anomalies of cardiac septal closure | 745 |
| Other congenital anomalies of heart | 746 |
| Other congenital anomalies of circulatory system | 747 |
| Congenital anomalies of respiratory system | 748 |
| [Cleft palate and cleft lip](http://eicd9.com/index.php?action=child&recordid=7524) | 749 |
| Other congenital anomalies of upper alimentary tract | 750 |
| Other congenital anomalies of digestive system | 751 |
| Genital Organs | 752 |
| Congenital anomalies of urinary system | 753 |
| Certain congenital musculoskeletal deformities | 754 |
| Other congenital anomalies of limbs | 755 |
| Other congenital musculoskeletal anomalies | 756 |
| Congenital anomalies of the integument | 757 |
| Chromosomal anomalies | 758 |
| [Other and unspecified congenital anomalies](http://eicd9.com/index.php?action=child&recordid=7795) | 759 |
| *Obesity* | |
| Overweight (Body Mass Index (BMI)< 30) | 278.02 |
| Obesity (BMI >30) | 278.00 |
| BMI pediatric risk for obesity 85th < BMI > 97th percent | V85.53 |
| BMI pediatric obese greater than or equal to 97th percent | V85.54 |
| BMI pediatric overweight 97th < BMI < 99.9th percentile for age | V85.54 |
| *Asthma-like illness* | |
| Ac. Bronchiolitis due to other infectious organisms | 466.19 |
| Ac. Bronchiolitis due to respiratory syncytial virus (rsv) | 466.11 |
| Acute bronchiolitis | 466.10 |
| Acute bronchitis/bronchiolitis | 466.00 |
| Asthma | V17.5 |
| Asthma routine follow up | V67 |
| Asthma, unspecified | 493.90 |
| Bronchial asthma | 493.9 |
| Bronchiolitis acute | 466.10 |
| Bronchitis acute fibrinous | 466.00 |
| Bronchitis asthmatic | *493.90* |
| Wheezing | 786.07 |
| Wheezing baby syndrome | 4651.00 |
| *Behavioral/developmental disorders* | |
| Attention deficit disorder with hyperactivity | 314.01 |
| Attention for observation | 7.00 |
| Autism spectrum disorder | 299.00 |
| *Malignancies* | |
| Burkitts lymphoma | 200.20 |
| Malignant neoplasm of kidney, except pelvis | 223.00 |

^1^ None of the cardiovascular outcomes was defined based on diagnosis, but solely on children' visits to cardiovascular clinics.
