# Supplementary material for: Maternal metal concentration during gestation and pediatric morbidity in children: an exploratory analysis
Source: Environ Health Prev Med. 2021 Mar 25;26:40. doi: 10.1186/s12199-021-00963-z (PMC7995788; doi:10.1186/s12199-021-00963-z)
Supplement: Supplementary file 3 — Additional file 3: Table S3: Association between maternal and newborn clinical background and presence of a clinical outcome at birth [file 12199_2021_963_MOESM3_ESM.rtf]

Table 3. Association between maternal and newborn clinical background and presence of a clinical outcome at birth 

Predicted outcome 	Covariate	Prevalence Ratio	95%CI (PR)	
Preterm delivery
	Maternal age, years	1.10	0.98; 1.22	
	Parity (1/2-5/6+)	0.63	0.26; 1.54	
	Male gender	0.57	0.20; 1.62	
Asthma	Maternal age, years	1.01	0.97; 1.04	
	Parity (1/2-5/6+)	0.87	0.67; 1.14	
	Male gender	1.06	0.83; 1.36	
	Preterm	0.95	0.63; 1.43	
Cardiovascular	Maternal age, years	1.10	0.98; 1.23	
	Parity (1/2-5/6+)	0.45	0.19; 1.07	
	Male gender	1.15	0.39; 3.41	
	Preterm	0.67	0.08; 5.62	
Behavioral or developmental	Maternal age, years	0.93	0.69; 1.26	
	Parity (1/2-5/6+)	1.01	0.11; 9.62	
	Male gender	6.69	0.64; 69.55	
	Preterm	4.99	0.96; 25.89	
Obesity	Maternal age, years	1.06	0.95; 1.17	
	Parity (1/2-5/6+)	0.66	0.18; 2.47	
	Male gender	0.85	0.16; 4.37	
	Preterm	1.46	0.14; 14.96	
Malformations	Maternal age, years	1.02	0.87; 1.19	
	Parity (1/2-5/6+)	1.01	0.31; 3.30	
	Male gender	1.36	0.31; 5.85	
	Preterm	1.15	0.13; 10.56	
Skin Diseases	Maternal age, years	1.05	1.02; 1.08	
	Parity (1/2-5/6+)	0.63	0.47; 0.84	
	Male gender	1.03	0.77; 1.38	
	Preterm	0.90	0.56; 1.44	
