# Supplementary figures and images for: Maternal metal concentration during gestation and pediatric morbidity in children: an exploratory analysis
Source: Environ Health Prev Med. 2021 Mar 25;26:40. doi: 10.1186/s12199-021-00963-z (PMC7995788; doi:10.1186/s12199-021-00963-z)

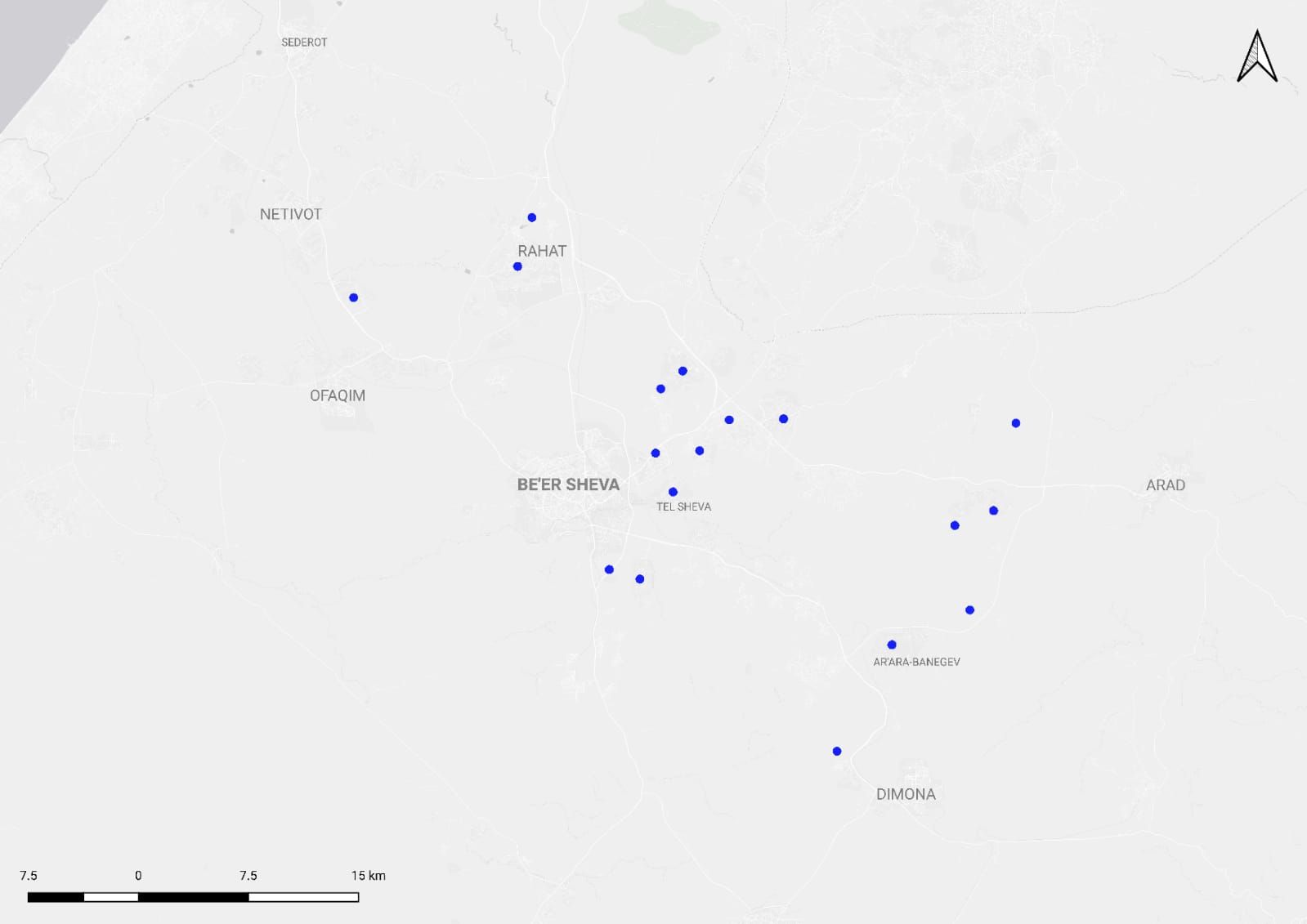

Supplement: Supplementary file 4 — Additional file 4: Figure S1: Study area map and geographical distribution of the study population. Figure legend: the blue dots in the map represent the subjects' residence location. (JPEG 45 kb) [file 12199_2021_963_MOESM4_ESM.jpeg]

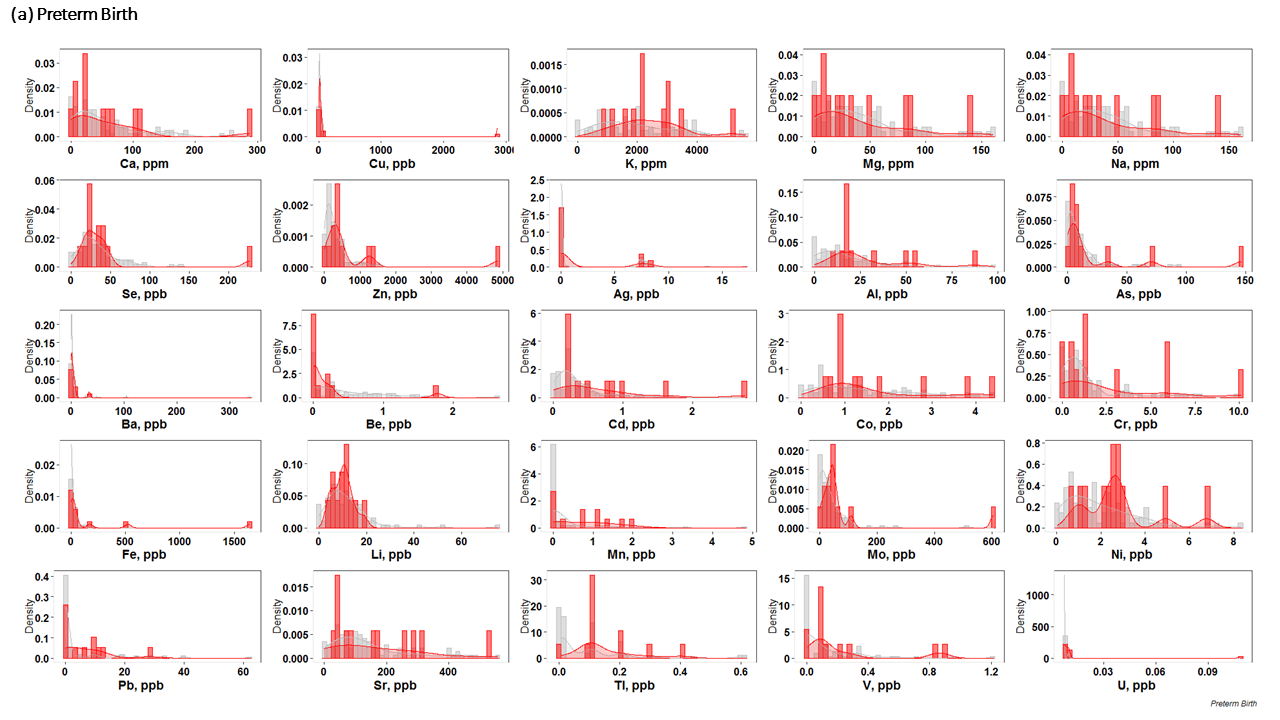

Supplement: Supplementary file 5 — Additional file 5: Figure S2: Graphical presentation of the metal concentrations by outcomes [file 12199_2021_963_MOESM5_ESM.zip › SuppMat_fig2a_preterm.tif]

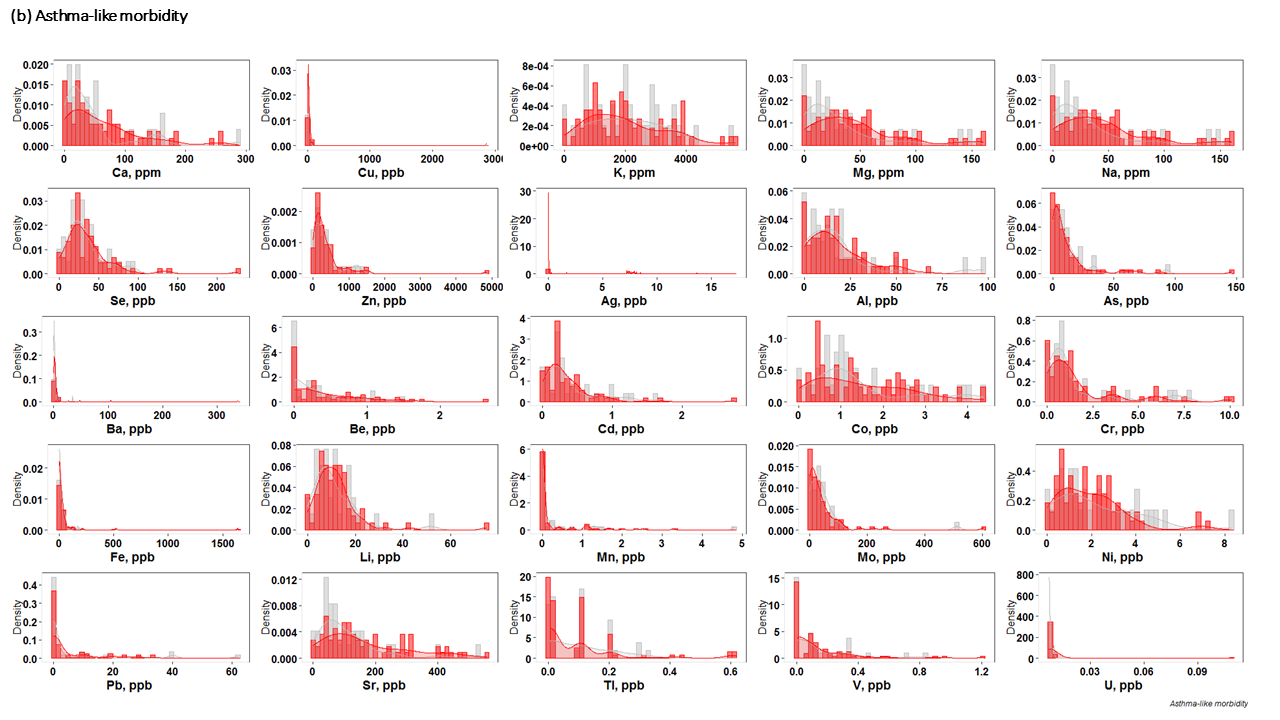

Supplement: Supplementary file 5 — Additional file 5: Figure S2: Graphical presentation of the metal concentrations by outcomes [file 12199_2021_963_MOESM5_ESM.zip › SuppMat_fig2b_asthma.tif]

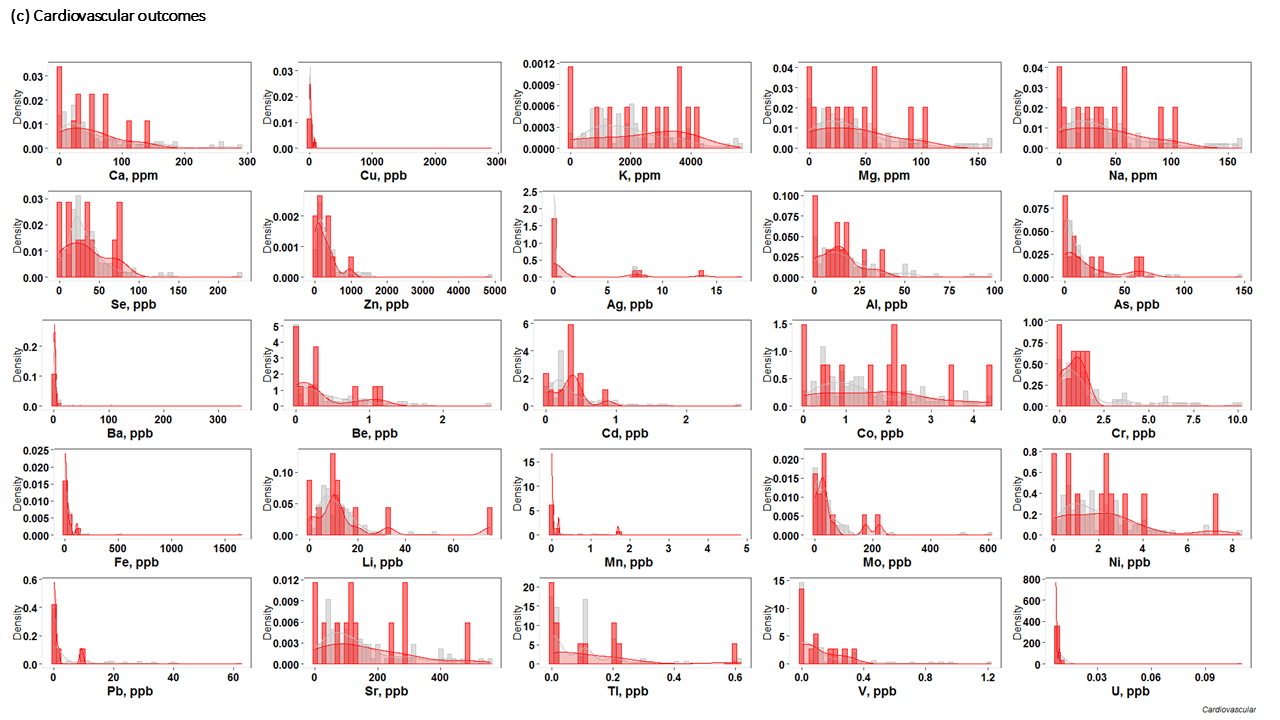

Supplement: Supplementary file 5 — Additional file 5: Figure S2: Graphical presentation of the metal concentrations by outcomes [file 12199_2021_963_MOESM5_ESM.zip › SuppMat_fig2c_cardio.tif]

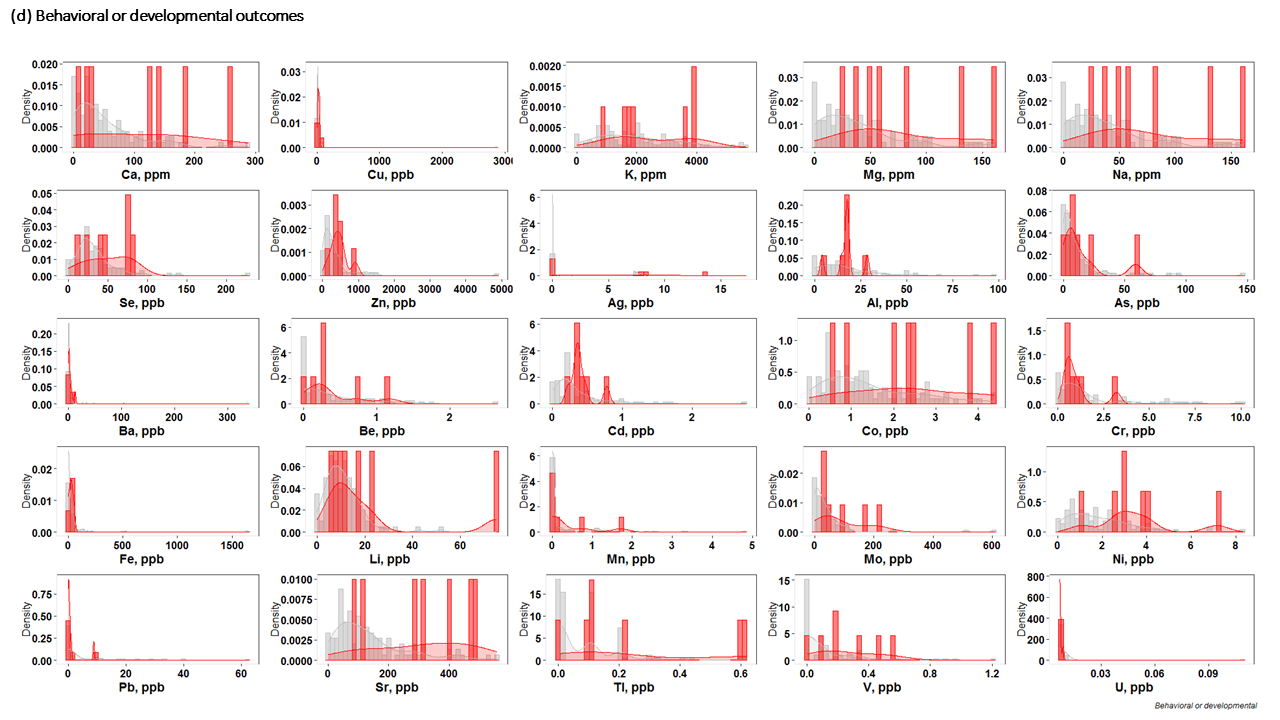

Supplement: Supplementary file 5 — Additional file 5: Figure S2: Graphical presentation of the metal concentrations by outcomes [file 12199_2021_963_MOESM5_ESM.zip › SuppMat_fig2d_behav.tif]

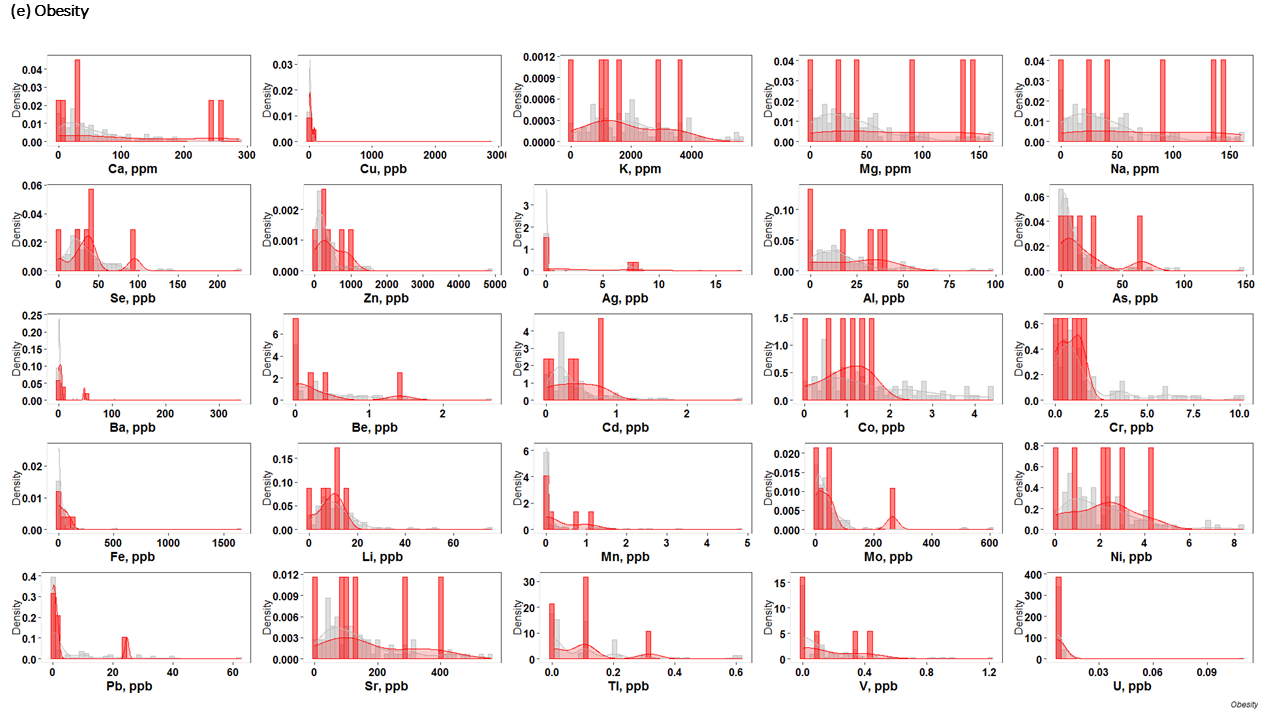

Supplement: Supplementary file 5 — Additional file 5: Figure S2: Graphical presentation of the metal concentrations by outcomes [file 12199_2021_963_MOESM5_ESM.zip › SuppMat_fig2e_obesity.tif]

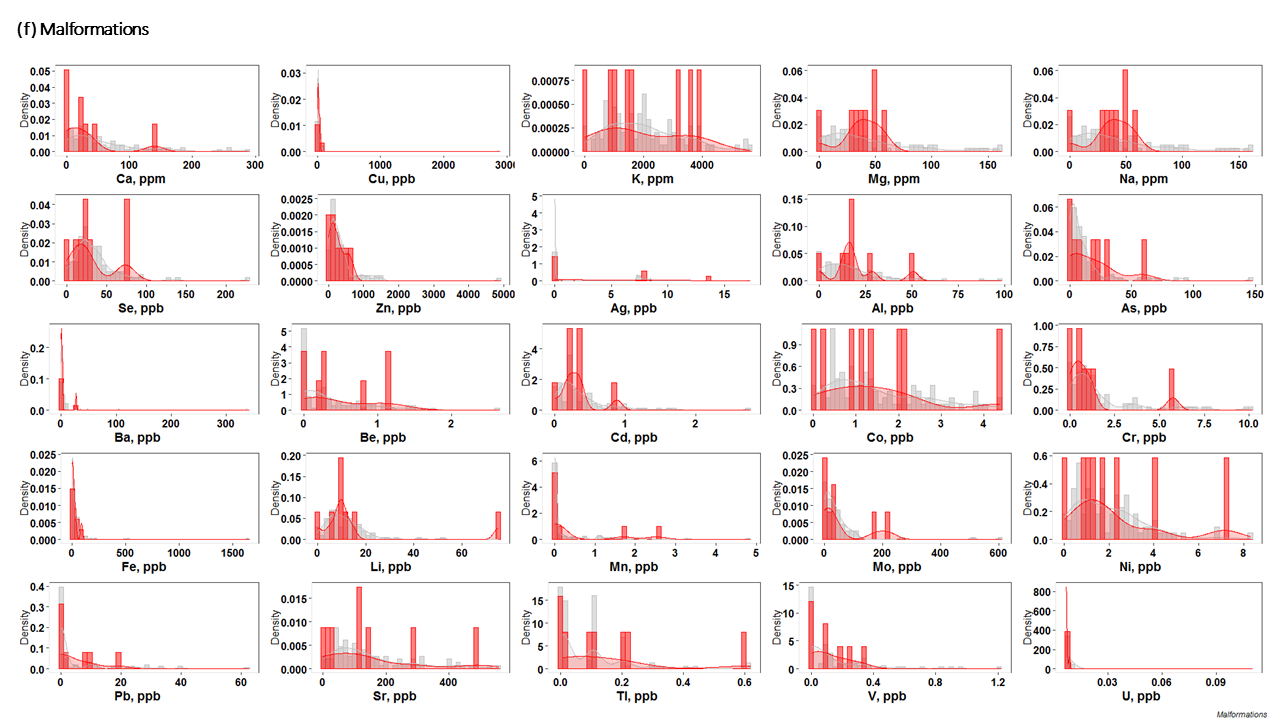

Supplement: Supplementary file 5 — Additional file 5: Figure S2: Graphical presentation of the metal concentrations by outcomes [file 12199_2021_963_MOESM5_ESM.zip › SuppMat_fig2f_malf.tif]
