# Supplementary material for: Maternal metal concentration during gestation and pediatric morbidity in children: an exploratory analysis
Source: Environ Health Prev Med. 2021 Mar 25;26:40. doi: 10.1186/s12199-021-00963-z (PMC7995788; doi:10.1186/s12199-021-00963-z)
Supplement: Supplementary file 6 — Additional file 6. [file 12199_2021_963_MOESM6_ESM.docx]

/******************************************************************************/

/* Project : MCM - FU

/* Program : FU_analysis.sas

/* Location :

/* Description : prenatal exposure to Heavy Metals and their association with pediatric morbidity

/* Modified by : Lena Novack

/* Date Created : Nov 21, 2020

/******************************************************************************/

OPTIONS MSGLEVEL=i NODATE NONUMBER NOFMTERR SOURCE NOTES NOXWAIT;

options yearcutoff=**1920**;

**%macro** sort (lib, dsn, var);

data &dsn;

set &lib.**.**&dsn;

run;

proc sort data=&dsn out=&dsn;

by &var;

run;

**%mend** sort;

libname outdata ".........";

%***sort***(outdata,data,code);

/*add quintiles*/;

**proc** **rank** data=labs_FU1 out=labs_FU1_ranked5 ties=low

groups=**5**; _

var Na_ppm K_ppm Mg_ppm Ca_ppm Se_ppb Zn_ppb Cu_ppb

Li_ppb Co_ppb Ni_ppb Tl_ppb Al_ppb Cr_ppb Sr_ppb Ba_ppb Cd_ppb Be_ppb V_ppb As_ppb Fe_ppb Mo_ppb Mn_ppb Ag_ppb Pb_ppb U_ppb;

ranks Na_r5 K_r5 Mg_r5 Ca_r5 Se_r5 Zn_r5 Cu_r5

Li_r5 Co_r5 Ni_r5 Tl_r5 Al_r5 Cr_r5 Sr_r5 Ba_r5 Cd_r5 Be_r5 V_r5 As_r5 Fe_r5 Mo_r5 Mn_r5 Ag_r5 Pb_r5 U_r5

;

**run**;

/*table 1*/

**proc** **freq** data=labs_FU1;

table age parity_3gr gestage_wk InfantWeigth SGA male;

table preterm anom01_B_FU /*malformations*/ major minor disRA /*asthma*/ disC /*cardiovascular*/ disB /*behavioural*/ disO /*obesity*/ BOD /*burden of disease*/;

**run**;

/*table 2 - metals' concentrations*/

**proc** **means** data=labs_FU1 n mean std min max p20 p40 p60 p80; where clalit=**1** and HUJIlab=**1**;

var Ca_ppm Cu_ppb K_ppm Mg_ppm Na_ppm Se_ppb Zn_ppb

Ag_ppb Al_ppb As_ppb Ba_ppb Be_ppb

Cd_ppb Co_ppb Cr_ppb Fe_ppb LI_ppb

Mn_ppb Mo_ppb Ni_ppb Pb_ppb Sr_ppb

Tl_ppb V_ppb U_ppb;

**run**;

**PROC** **SURVEYMEANS** data=labs_FU1 geomean GMCLM;where clalit=**1** and HUJIlab=**1**;

var Ca_ppm Cu_ppb K_ppm Mg_ppm Na_ppm Se_ppb Zn_ppb

Ag_ppb Al_ppb As_ppb Ba_ppb Be_ppb

Cd_ppb Co_ppb Cr_ppb Fe_ppb LI_ppb

Mn_ppb Mo_ppb Ni_ppb Pb_ppb Sr_ppb

Tl_ppb V_ppb U_ppb;

**run**;

/*link between metals and morbidities*/;

**data** labs_FU2; set labs_FU1; if clalit=**1** and HUJIlab=**1**; **run**;

/*univariable analysis*/

**%macro** ttest(ds,metal,factor, ds_new, order, metal_ln, model_ds);

proc ttest data=&ds dist=lognormal test=ratio; var &metal; class &factor;

ods output Statistics=&ds_new (keep=n class geomMean LowerCLGeomMean UpperCLGeomMean); run;

data &ds_new; set &ds_new; var="&factor";order=&order; if class^="Ratio (1/2)"; run;

proc reg data = &ds; model &metal_ln = &factor;

ods output ParameterEstimates=&model_ds (keep=Dependent Variable estimate stdErr probt); run;

data &model_ds; set &model_ds; if variable^="Intercept"; order=&order; run;

**%mend** ttest;

/*explore univariable association between metals and pediatric morbidity*/

/*Na*/

%***ttest*** (labs_FU2, Na_ppm, disRA, GM_Na_disRA, **1**, Na_ppm_ln, mod_Na_disRA);

%***ttest*** (labs_FU2, Na_ppm, disC, GM_Na_disC, **3**, Na_ppm_ln, mod_Na_disC);

%***ttest*** (labs_FU2, Na_ppm, disB, GM_Na_disB, **4**, Na_ppm_ln, mod_Na_disB);

%***ttest*** (labs_FU2, Na_ppm, disO, GM_Na_disO, **5**, Na_ppm_ln, mod_Na_disO);

%***ttest*** (labs_FU2, Na_ppm, anom01_B_FU, GM_Na_anom01_B_FU, **8**, Na_ppm_ln, mod_Na_anom01_B_FU);

**data** GMs_Na_bydis; set GM_Na_disRA GM_Na_disC GM_Na_disB GM_Na_disO GM_Na_anom01_B_FU;

format geomMean LowerCLGeomMean UpperCLGeomMean **8.2**;

**run**;

**data** Ratios_Na; set mod_Na_disRA mod_Na_disC mod_Na_disB mod_Na_disO mod_Na_anom01_B_FU;

Exp_est=exp(estimate);

LL=exp(estimate-**1.96***StdErr);

UL=exp(estimate+**1.96***StdErr);

keep order variable Exp_est LL UL Probt class;

format Exp_est LL UL **8.2**; format Probt **5.3**;

**run**;

%***sort***(work,GMs_Na_bydis, order);

%***sort***(work,Ratios_Na, order);

**data** Metals_desc_Na; merge GMs_Na_bydis Ratios_Na; by order; **run**;

/*K*/

%***ttest*** (labs_FU2, K_ppm, disRA, GM_K_disRA, **1**, K_ppm_ln, mod_K_disRA);

%***ttest*** (labs_FU2, K_ppm, disC, GM_K_disC, **3**, K_ppm_ln, mod_K_disC);

%***ttest*** (labs_FU2, K_ppm, disB, GM_K_disB, **4**, K_ppm_ln, mod_K_disB);

%***ttest*** (labs_FU2, K_ppm, disO, GM_K_disO, **5**, K_ppm_ln, mod_K_disO);

%***ttest*** (labs_FU2, K_ppm, anom01_B_FU, GM_K_anom01_B_FU, **8**, K_ppm_ln, mod_K_anom01_B_FU);

**data** GMs_K_bydis; set GM_K_disRA GM_K_disC GM_K_disB GM_K_disO GM_K_anom01_B_FU;

format geomMean LowerCLGeomMean UpperCLGeomMean **8.2**;

**run**;

**data** Ratios_K; set mod_K_disRA mod_K_disC mod_K_disB mod_K_disO mod_K_anom01_B_FU;

Exp_est=exp(estimate);

LL=exp(estimate-**1.96***StdErr);

UL=exp(estimate+**1.96***StdErr);

keep order variable Exp_est LL UL Probt class;

format Exp_est LL UL **8.2**; format Probt **5.3**;

**run**;

%***sort***(work,GMs_K_bydis, order);

%***sort***(work,Ratios_K, order);

**data** Metals_desc_K; merge GMs_K_bydis Ratios_K; by order; **run**;

/*Mg*/

%***ttest*** (labs_FU2, Mg_ppm, disRA, GM_Mg_disRA, **1**, Mg_ppm_ln, mod_Mg_disRA);

%***ttest*** (labs_FU2, Mg_ppm, disC, GM_Mg_disC, **3**, Mg_ppm_ln, mod_Mg_disC);

%***ttest*** (labs_FU2, Mg_ppm, disB, GM_Mg_disB, **4**, Mg_ppm_ln, mod_Mg_disB);

%***ttest*** (labs_FU2, Mg_ppm, disO, GM_Mg_disO, **5**, Mg_ppm_ln, mod_Mg_disO);

%***ttest*** (labs_FU2, Mg_ppm, anom01_B_FU, GM_Mg_anom01_B_FU, **8**, Mg_ppm_ln, mod_Mg_anom01_B_FU);

**data** GMs_Mg_bydis; set GM_Mg_disRA GM_Mg_disC GM_Mg_disB GM_Mg_disO GM_Mg_anom01_B_FU;

format geomMean LowerCLGeomMean UpperCLGeomMean **8.2**;

**run**;

**data** Ratios_Mg; set mod_Mg_disRA mod_Mg_disC mod_Mg_disB mod_Mg_disO mod_Mg_anom01_B_FU;

Exp_est=exp(estimate);

LL=exp(estimate-**1.96***StdErr);

UL=exp(estimate+**1.96***StdErr);

keep order variable Exp_est LL UL Probt class;

format Exp_est LL UL **8.2**; format Probt **5.3**;

**run**;

%***sort***(work,GMs_Mg_bydis, order);

%***sort***(work,Ratios_Mg, order);

**data** Metals_desc_Mg; merge GMs_Mg_bydis Ratios_Mg; by order; **run**;

/*Ca*/

%***ttest*** (labs_FU2, Ca_ppm, disRA, GM_Ca_disRA, **1**, Ca_ppm_ln, mod_Ca_disRA);

%***ttest*** (labs_FU2, Ca_ppm, disC, GM_Ca_disC, **3**, Ca_ppm_ln, mod_Ca_disC);

%***ttest*** (labs_FU2, Ca_ppm, disB, GM_Ca_disB, **4**, Ca_ppm_ln, mod_Ca_disB);

%***ttest*** (labs_FU2, Ca_ppm, disO, GM_Ca_disO, **5**, Ca_ppm_ln, mod_Ca_disO);

%***ttest*** (labs_FU2, Ca_ppm, anom01_B_FU, GM_Ca_anom01_B_FU, **8**, Ca_ppm_ln, mod_Ca_anom01_B_FU);

**data** GMs_Ca_bydis; set GM_Ca_disRA GM_Ca_disC GM_Ca_disB GM_Ca_disO GM_Ca_anom01_B_FU;

format geomMean LowerCLGeomMean UpperCLGeomMean **8.2**;

**run**;

**data** Ratios_Ca; set mod_Ca_disRA mod_Ca_disC mod_Ca_disB mod_Ca_disO mod_Ca_anom01_B_FU;

Exp_est=exp(estimate);

LL=exp(estimate-**1.96***StdErr);

UL=exp(estimate+**1.96***StdErr);

keep order variable Exp_est LL UL Probt class;

format Exp_est LL UL **8.2**; format Probt **5.3**;

**run**;

%***sort***(work,GMs_Ca_bydis, order);

%***sort***(work,Ratios_Ca, order);

**data** Metals_desc_Ca; merge GMs_Ca_bydis Ratios_Ca; by order; **run**;

/*Se*/

%***ttest*** (labs_FU2, Se_ppb, disRA, GM_Se_disRA, **1**, Se_ppb_ln, mod_Se_disRA);

%***ttest*** (labs_FU2, Se_ppb, disC, GM_Se_disC, **3**, Se_ppb_ln, mod_Se_disC);

%***ttest*** (labs_FU2, Se_ppb, disB, GM_Se_disB, **4**, Se_ppb_ln, mod_Se_disB);

%***ttest*** (labs_FU2, Se_ppb, disO, GM_Se_disO, **5**, Se_ppb_ln, mod_Se_disO);

%***ttest*** (labs_FU2, Se_ppb, anom01_B_FU, GM_Se_anom01_B_FU, **8**, Se_ppb_ln, mod_Se_anom01_B_FU);

**data** GMs_Se_bydis; set GM_Se_disRA GM_Se_disC GM_Se_disB GM_Se_disO GM_Se_anom01_B_FU;

format geomMean LowerCLGeomMean UpperCLGeomMean **8.2**;

**run**;

**data** Ratios_Se; set mod_Se_disRA mod_Se_disC mod_Se_disB mod_Se_disO mod_Se_anom01_B_FU;

Exp_est=exp(estimate);

LL=exp(estimate-**1.96***StdErr);

UL=exp(estimate+**1.96***StdErr);

keep order variable Exp_est LL UL Probt class;

format Exp_est LL UL **8.2**; format Probt **5.3**;

**run**;

%***sort***(work,GMs_Se_bydis, order);

%***sort***(work,Ratios_Se, order);

**data** Metals_desc_Se; merge GMs_Se_bydis Ratios_Se; by order; **run**;

/*Zn*/

%***ttest*** (labs_FU2, Zn_ppb, disRA, GM_Zn_disRA, **1**, Zn_ppb_ln, mod_Zn_disRA);

%***ttest*** (labs_FU2, Zn_ppb, disC, GM_Zn_disC, **3**, Zn_ppb_ln, mod_Zn_disC);

%***ttest*** (labs_FU2, Zn_ppb, disB, GM_Zn_disB, **4**, Zn_ppb_ln, mod_Zn_disB);

%***ttest*** (labs_FU2, Zn_ppb, disO, GM_Zn_disO, **5**, Zn_ppb_ln, mod_Zn_disO);

%***ttest*** (labs_FU2, Zn_ppb, anom01_B_FU, GM_Zn_anom01_B_FU, **8**, Zn_ppb_ln, mod_Zn_anom01_B_FU);

**data** GMs_Zn_bydis; set GM_Zn_disRA GM_Zn_disC GM_Zn_disB GM_Zn_disO GM_Zn_anom01_B_FU;

format geomMean LowerCLGeomMean UpperCLGeomMean **8.2**;

**run**;

**data** Ratios_Zn; set mod_Zn_disRA mod_Zn_disC mod_Zn_disB mod_Zn_disO mod_Zn_anom01_B_FU;

Exp_est=exp(estimate);

LL=exp(estimate-**1.96***StdErr);

UL=exp(estimate+**1.96***StdErr);

keep order variable Exp_est LL UL Probt class;

format Exp_est LL UL **8.2**; format Probt **5.3**;

**run**;

%***sort***(work,GMs_Zn_bydis, order);

%***sort***(work,Ratios_Zn, order);

**data** Metals_desc_Zn; merge GMs_Zn_bydis Ratios_Zn; by order; **run**;

/*Cu*/

%***ttest*** (labs_FU2, Cu_ppb, disRA, GM_Cu_disRA, **1**, Cu_ppb_ln, mod_Cu_disRA);

%***ttest*** (labs_FU2, Cu_ppb, disC, GM_Cu_disC, **3**, Cu_ppb_ln, mod_Cu_disC);

%***ttest*** (labs_FU2, Cu_ppb, disB, GM_Cu_disB, **4**, Cu_ppb_ln, mod_Cu_disB);

%***ttest*** (labs_FU2, Cu_ppb, disO, GM_Cu_disO, **5**, Cu_ppb_ln, mod_Cu_disO);

%***ttest*** (labs_FU2, Cu_ppb, anom01_B_FU, GM_Cu_anom01_B_FU, **8**, Cu_ppb_ln, mod_Cu_anom01_B_FU);

**data** GMs_Cu_bydis; set GM_Cu_disRA GM_Cu_disC GM_Cu_disB GM_Cu_disO GM_Cu_anom01_B_FU;

format geomMean LowerCLGeomMean UpperCLGeomMean **8.2**;

**run**;

**data** Ratios_Cu; set mod_Cu_disRA mod_Cu_disC mod_Cu_disB mod_Cu_disO mod_Cu_anom01_B_FU;

Exp_est=exp(estimate);

LL=exp(estimate-**1.96***StdErr);

UL=exp(estimate+**1.96***StdErr);

keep order variable Exp_est LL UL Probt class;

format Exp_est LL UL **8.2**; format Probt **5.3**;

**run**;

%***sort***(work,GMs_Cu_bydis, order);

%***sort***(work,Ratios_Cu, order);

**data** Metals_desc_Cu; merge GMs_Cu_bydis Ratios_Cu; by order; **run**;

/*Li*/

%***ttest*** (labs_FU2, Li_ppb, disRA, GM_Li_disRA, **1**, Li_ppb_ln, mod_Li_disRA);

%***ttest*** (labs_FU2, Li_ppb, disC, GM_Li_disC, **3**, Li_ppb_ln, mod_Li_disC);

%***ttest*** (labs_FU2, Li_ppb, disB, GM_Li_disB, **4**, Li_ppb_ln, mod_Li_disB);

%***ttest*** (labs_FU2, Li_ppb, disO, GM_Li_disO, **5**, Li_ppb_ln, mod_Li_disO);

%***ttest*** (labs_FU2, Li_ppb, anom01_B_FU, GM_Li_anom01_B_FU, **8**, Li_ppb_ln, mod_Li_anom01_B_FU);

**data** GMs_Li_bydis; set GM_Li_disRA GM_Li_disC GM_Li_disB GM_Li_disO GM_Li_anom01_B_FU;

format geomMean LowerCLGeomMean UpperCLGeomMean **8.2**;

**run**;

**data** Ratios_Li; set mod_Li_disRA mod_Li_disC mod_Li_disB mod_Li_disO mod_Li_anom01_B_FU;

Exp_est=exp(estimate);

LL=exp(estimate-**1.96***StdErr);

UL=exp(estimate+**1.96***StdErr);

keep order variable Exp_est LL UL Probt class;

format Exp_est LL UL **8.2**; format Probt **5.3**;

**run**;

%***sort***(work,GMs_Li_bydis, order);

%***sort***(work,Ratios_Li, order);

**data** Metals_desc_Li; merge GMs_Li_bydis Ratios_Li; by order; **run**;

/*Co*/

%***ttest*** (labs_FU2, Co_ppb, disRA, GM_Co_disRA, **1**, Co_ppb_ln, mod_Co_disRA);

%***ttest*** (labs_FU2, Co_ppb, disC, GM_Co_disC, **3**, Co_ppb_ln, mod_Co_disC);

%***ttest*** (labs_FU2, Co_ppb, disB, GM_Co_disB, **4**, Co_ppb_ln, mod_Co_disB);

%***ttest*** (labs_FU2, Co_ppb, disO, GM_Co_disO, **5**, Co_ppb_ln, mod_Co_disO);

%***ttest*** (labs_FU2, Co_ppb, anom01_B_FU, GM_Co_anom01_B_FU, **8**, Co_ppb_ln, mod_Co_anom01_B_FU);

**data** GMs_Co_bydis; set GM_Co_disRAGM_Co_disC GM_Co_disB GM_Co_disO GM_Co_anom01_B_FU;

format geomMean LowerCLGeomMean UpperCLGeomMean **8.2**;

**run**;

**data** Ratios_Co; set mod_Co_disRA mod_Co_disC mod_Co_disB mod_Co_disO mod_Co_anom01_B_FU;

Exp_est=exp(estimate);

LL=exp(estimate-**1.96***StdErr);

UL=exp(estimate+**1.96***StdErr);

keep order variable Exp_est LL UL Probt class;

format Exp_est LL UL **8.2**; format Probt **5.3**;

**run**;

%***sort***(work,GMs_Co_bydis, order);

%***sort***(work,Ratios_Co, order);

**data** Metals_desc_Co; merge GMs_Co_bydis Ratios_Co; by order; **run**;

/*Ni*/

%***ttest*** (labs_FU2, Ni_ppb, disRA, GM_Ni_disRA, **1**, Ni_ppb_ln, mod_Ni_disRA);

%***ttest*** (labs_FU2, Ni_ppb, disC, GM_Ni_disC, **3**, Ni_ppb_ln, mod_Ni_disC);

%***ttest*** (labs_FU2, Ni_ppb, disB, GM_Ni_disB, **4**, Ni_ppb_ln, mod_Ni_disB);

%***ttest*** (labs_FU2, Ni_ppb, disO, GM_Ni_disO, **5**, Ni_ppb_ln, mod_Ni_disO);

%***ttest*** (labs_FU2, Ni_ppb, anom01_B_FU, GM_Ni_anom01_B_FU, **8**, Ni_ppb_ln, mod_Ni_anom01_B_FU);

**data** GMs_Ni_bydis; set GM_Ni_disRA GM_Ni_disC GM_Ni_disB GM_Ni_disO GM_Ni_anom01_B_FU;

format geomMean LowerCLGeomMean UpperCLGeomMean **8.2**;

**run**;

**data** Ratios_Ni; set mod_Ni_disRA mod_Ni_disC mod_Ni_disB mod_Ni_disO mod_Ni_anom01_B_FU;

Exp_est=exp(estimate);

LL=exp(estimate-**1.96***StdErr);

UL=exp(estimate+**1.96***StdErr);

keep order variable Exp_est LL UL Probt class;

format Exp_est LL UL **8.2**; format Probt **5.3**;

**run**;

%***sort***(work,GMs_Ni_bydis, order);

%***sort***(work,Ratios_Ni, order);

**data** Metals_desc_Ni; merge GMs_Ni_bydis Ratios_Ni; by order; **run**;

/*Tl*/

%***ttest*** (labs_FU2, Tl_ppb, disRA, GM_Tl_disRA, **1**, Tl_ppb_ln, mod_Tl_disRA);

%***ttest*** (labs_FU2, Tl_ppb, disC, GM_Tl_disC, **3**, Tl_ppb_ln, mod_Tl_disC);

%***ttest*** (labs_FU2, Tl_ppb, disB, GM_Tl_disB, **4**, Tl_ppb_ln, mod_Tl_disB);

%***ttest*** (labs_FU2, Tl_ppb, disO, GM_Tl_disO, **5**, Tl_ppb_ln, mod_Tl_disO);

%***ttest*** (labs_FU2, Tl_ppb, anom01_B_FU, GM_Tl_anom01_B_FU, **8**, Tl_ppb_ln, mod_Tl_anom01_B_FU);

**data** GMs_Tl_bydis; set GM_Tl_disRA GM_Tl_disC GM_Tl_disB GM_Tl_disO GM_Tl_anom01_B_FU;

format geomMean LowerCLGeomMean UpperCLGeomMean **8.2**;

**run**;

**data** Ratios_Tl; set mod_Tl_disRA mod_Tl_disC mod_Tl_disB mod_Tl_disO mod_Tl_anom01_B_FU;

Exp_est=exp(estimate);

LL=exp(estimate-**1.96***StdErr);

UL=exp(estimate+**1.96***StdErr);

keep order variable Exp_est LL UL Probt class;

format Exp_est LL UL **8.2**; format Probt **5.3**;

**run**;

%***sort***(work,GMs_Tl_bydis, order);

%***sort***(work,Ratios_Tl, order);

**data** Metals_desc_Tl; merge GMs_Tl_bydis Ratios_Tl; by order; **run**;

/*Al*/

%***ttest*** (labs_FU2, Al_ppb, disRA, GM_Al_disRA, **1**, Al_ppb_ln, mod_Al_disRA);

%***ttest*** (labs_FU2, Al_ppb, disC, GM_Al_disC, **3**, Al_ppb_ln, mod_Al_disC);

%***ttest*** (labs_FU2, Al_ppb, disB, GM_Al_disB, **4**, Al_ppb_ln, mod_Al_disB);

%***ttest*** (labs_FU2, Al_ppb, disO, GM_Al_disO, **5**, Al_ppb_ln, mod_Al_disO);

%***ttest*** (labs_FU2, Al_ppb, anom01_B_FU, GM_Al_anom01_B_FU, **8**, Al_ppb_ln, mod_Al_anom01_B_FU);

**data** GMs_Al_bydis; set GM_Al_disRA GM_Al_disC GM_Al_disB GM_Al_disO GM_Al_anom01_B_FU;

format geomMean LowerCLGeomMean UpperCLGeomMean **8.2**;

**run**;

**data** Ratios_Al; set mod_Al_disRA mod_Al_disC mod_Al_disB mod_Al_disO mod_Al_anom01_B_FU;

Exp_est=exp(estimate);

LL=exp(estimate-**1.96***StdErr);

UL=exp(estimate+**1.96***StdErr);

keep order variable Exp_est LL UL Probt class;

format Exp_est LL UL **8.2**; format Probt **5.3**;

**run**;

%***sort***(work,GMs_Al_bydis, order);

%***sort***(work,Ratios_Al, order);

**data** Metals_desc_Al; merge GMs_Al_bydis Ratios_Al; by order; **run**;

/*Cr*/

%***ttest*** (labs_FU2, Cr_ppb, disRA, GM_Cr_disRA, **1**, Cr_ppb_ln, mod_Cr_disRA);

%***ttest*** (labs_FU2, Cr_ppb, disC, GM_Cr_disC, **3**, Cr_ppb_ln, mod_Cr_disC);

%***ttest*** (labs_FU2, Cr_ppb, disB, GM_Cr_disB, **4**, Cr_ppb_ln, mod_Cr_disB);

%***ttest*** (labs_FU2, Cr_ppb, disO, GM_Cr_disO, **5**, Cr_ppb_ln, mod_Cr_disO);

%***ttest*** (labs_FU2, Cr_ppb, anom01_B_FU, GM_Cr_anom01_B_FU, **8**, Cr_ppb_ln, mod_Cr_anom01_B_FU);

**data** GMs_Cr_bydis; set GM_Cr_disRA GM_Cr_disC GM_Cr_disB GM_Cr_disO GM_Cr_anom01_B_FU;

format geomMean LowerCLGeomMean UpperCLGeomMean **8.2**;

**run**;

**data** Ratios_Cr; set mod_Cr_disRA mod_Cr_disC mod_Cr_disB mod_Cr_disO mod_Cr_anom01_B_FU;

Exp_est=exp(estimate);

LL=exp(estimate-**1.96***StdErr);

UL=exp(estimate+**1.96***StdErr);

keep order variable Exp_est LL UL Probt class;

format Exp_est LL UL **8.2**; format Probt **5.3**;

**run**;

%***sort***(work,GMs_Cr_bydis, order);

%***sort***(work,Ratios_Cr, order);

**data** Metals_desc_Cr; merge GMs_Cr_bydis Ratios_Cr; by order; **run**;

/*Sr*/

%***ttest*** (labs_FU2, Sr_ppb, disRA, GM_Sr_disRA, **1**, Sr_ppb_ln, mod_Sr_disRA);

%***ttest*** (labs_FU2, Sr_ppb, disC, GM_Sr_disC, **3**, Sr_ppb_ln, mod_Sr_disC);

%***ttest*** (labs_FU2, Sr_ppb, disB, GM_Sr_disB, **4**, Sr_ppb_ln, mod_Sr_disB);

%***ttest*** (labs_FU2, Sr_ppb, disO, GM_Sr_disO, **5**, Sr_ppb_ln, mod_Sr_disO);

%***ttest*** (labs_FU2, Sr_ppb, anom01_B_FU, GM_Sr_anom01_B_FU, **8**, Sr_ppb_ln, mod_Sr_anom01_B_FU);

**data** GMs_Sr_bydis; set GM_Sr_disRA GM_Sr_disC GM_Sr_disB GM_Sr_disO GM_Sr_anom01_B_FU;

format geomMean LowerCLGeomMean UpperCLGeomMean **8.2**;

**run**;

**data** Ratios_Sr; set mod_Sr_disRA mod_Sr_disC mod_Sr_disB mod_Sr_disO mod_Sr_anom01_B_FU;

Exp_est=exp(estimate);

LL=exp(estimate-**1.96***StdErr);

UL=exp(estimate+**1.96***StdErr);

keep order variable Exp_est LL UL Probt class;

format Exp_est LL UL **8.2**; format Probt **5.3**;

**run**;

%***sort***(work,GMs_Sr_bydis, order);

%***sort***(work,Ratios_Sr, order);

**data** Metals_desc_Sr; merge GMs_Sr_bydis Ratios_Sr; by order; **run**;

/*Ba*/

%***ttest*** (labs_FU2, Ba_ppb, disRA, GM_Ba_disRA, **1**, Ba_ppb_ln, mod_Ba_disRA);

%***ttest*** (labs_FU2, Ba_ppb, disC, GM_Ba_disC, **3**, Ba_ppb_ln, mod_Ba_disC);

%***ttest*** (labs_FU2, Ba_ppb, disB, GM_Ba_disB, **4**, Ba_ppb_ln, mod_Ba_disB);

%***ttest*** (labs_FU2, Ba_ppb, disO, GM_Ba_disO, **5**, Ba_ppb_ln, mod_Ba_disO);

%***ttest*** (labs_FU2, Ba_ppb, anom01_B_FU, GM_Ba_anom01_B_FU, **8**, Ba_ppb_ln, mod_Ba_anom01_B_FU);

**data** GMs_Ba_bydis; set GM_Ba_disRA GM_Ba_disC GM_Ba_disB GM_Ba_disO GM_Ba_anom01_B_FU;

format geomMean LowerCLGeomMean UpperCLGeomMean **8.2**;

**run**;

**data** Ratios_Ba; set mod_Ba_disRA mod_Ba_disC mod_Ba_disB mod_Ba_disO mod_Ba_anom01_B_FU;

Exp_est=exp(estimate);

LL=exp(estimate-**1.96***StdErr);

UL=exp(estimate+**1.96***StdErr);

keep order variable Exp_est LL UL Probt class;

format Exp_est LL UL **8.2**; format Probt **5.3**;

**run**;

%***sort***(work,GMs_Ba_bydis, order);

%***sort***(work,Ratios_Ba, order);

**data** Metals_desc_Ba; merge GMs_Ba_bydis Ratios_Ba; by order; **run**;

/*Cd*/

%***ttest*** (labs_FU2, Cd_ppb, disRA, GM_Cd_disRA, **1**, Cd_ppb_ln, mod_Cd_disRA);

%***ttest*** (labs_FU2, Cd_ppb, disC, GM_Cd_disC, **3**, Cd_ppb_ln, mod_Cd_disC);

%***ttest*** (labs_FU2, Cd_ppb, disB, GM_Cd_disB, **4**, Cd_ppb_ln, mod_Cd_disB);

%***ttest*** (labs_FU2, Cd_ppb, disO, GM_Cd_disO, **5**, Cd_ppb_ln, mod_Cd_disO);

%***ttest*** (labs_FU2, Cd_ppb, anom01_B_FU, GM_Cd_anom01_B_FU, **8**, Cd_ppb_ln, mod_Cd_anom01_B_FU);

**data** GMs_Cd_bydis; set GM_Cd_disRA GM_Cd_disRO GM_Cd_disC GM_Cd_disB GM_Cd_disO GM_Cd_disEye GM_Cd_disOrtho GM_Cd_anom01_B_FU;

format geomMean LowerCLGeomMean UpperCLGeomMean **8.2**;

**run**;

**data** Ratios_Cd; set mod_Cd_disRA mod_Cd_disC mod_Cd_disB mod_Cd_disO mod_Cd_anom01_B_FU;

Exp_est=exp(estimate);

LL=exp(estimate-**1.96***StdErr);

UL=exp(estimate+**1.96***StdErr);

keep order variable Exp_est LL UL Probt class;

format Exp_est LL UL **8.2**; format Probt **5.3**;

**run**;

%***sort***(work,GMs_Cd_bydis, order);

%***sort***(work,Ratios_Cd, order);

**data** Metals_desc_Cd; merge GMs_Cd_bydis Ratios_Cd; by order; **run**;

/*Be*/

%***ttest*** (labs_FU2, Be_ppb, disRA, GM_Be_disRA, **1**, Be_ppb_ln, mod_Be_disRA);

%***ttest*** (labs_FU2, Be_ppb, disC, GM_Be_disC, **3**, Be_ppb_ln, mod_Be_disC);

%***ttest*** (labs_FU2, Be_ppb, disB, GM_Be_disB, **4**, Be_ppb_ln, mod_Be_disB);

%***ttest*** (labs_FU2, Be_ppb, disO, GM_Be_disO, **5**, Be_ppb_ln, mod_Be_disO);

%***ttest*** (labs_FU2, Be_ppb, anom01_B_FU, GM_Be_anom01_B_FU, **8**, Be_ppb_ln, mod_Be_anom01_B_FU);

**data** GMs_Be_bydis; set GM_Be_disRA GM_Be_disC GM_Be_disB GM_Be_disO GM_Be_anom01_B_FU;

format geomMean LowerCLGeomMean UpperCLGeomMean **8.2**;

**run**;

**data** Ratios_Be; set mod_Be_disRA mod_Be_disC mod_Be_disB mod_Be_disO mod_Be_anom01_B_FU;

Exp_est=exp(estimate);

LL=exp(estimate-**1.96***StdErr);

UL=exp(estimate+**1.96***StdErr);

keep order variable Exp_est LL UL Probt class;

format Exp_est LL UL **8.2**; format Probt **5.3**;

**run**;

%***sort***(work,GMs_Be_bydis, order);

%***sort***(work,Ratios_Be, order);

**data** Metals_desc_Be; merge GMs_Be_bydis Ratios_Be; by order; **run**;

/*V*/

%***ttest*** (labs_FU2, V_ppb, disRA, GM_V_disRA, **1**, V_ppb_ln, mod_V_disRA);

%***ttest*** (labs_FU2, V_ppb, disC, GM_V_disC, **3**, V_ppb_ln, mod_V_disC);

%***ttest*** (labs_FU2, V_ppb, disB, GM_V_disB, **4**, V_ppb_ln, mod_V_disB);

%***ttest*** (labs_FU2, V_ppb, disO, GM_V_disO, **5**, V_ppb_ln, mod_V_disO);

%***ttest*** (labs_FU2, V_ppb, anom01_B_FU, GM_V_anom01_B_FU, **8**, V_ppb_ln, mod_V_anom01_B_FU);

**data** GMs_V_bydis; set GM_V_disRA GM_V_disC GM_V_disB GM_V_disO GM_V_anom01_B_FU;

format geomMean LowerCLGeomMean UpperCLGeomMean **8.2**;

**run**;

**data** Ratios_V; set mod_V_disRA mod_V_disC mod_V_disB mod_V_disO mod_V_anom01_B_FU;

Exp_est=exp(estimate);

LL=exp(estimate-**1.96***StdErr);

UL=exp(estimate+**1.96***StdErr);

keep order variable Exp_est LL UL Probt class;

format Exp_est LL UL **8.2**; format Probt **5.3**;

**run**;

%***sort***(work,GMs_V_bydis, order);

%***sort***(work,Ratios_V, order);

**data** Metals_desc_V; merge GMs_V_bydis Ratios_V; by order; **run**;

/*As*/

%***ttest*** (labs_FU2, As_ppb, disRA, GM_As_disRA, **1**, As_ppb_ln, mod_As_disRA);

%***ttest*** (labs_FU2, As_ppb, disC, GM_As_disC, **3**, As_ppb_ln, mod_As_disC);

%***ttest*** (labs_FU2, As_ppb, disB, GM_As_disB, **4**, As_ppb_ln, mod_As_disB);

%***ttest*** (labs_FU2, As_ppb, disO, GM_As_disO, **5**, As_ppb_ln, mod_As_disO);

%***ttest*** (labs_FU2, As_ppb, anom01_B_FU, GM_As_anom01_B_FU, **8**, As_ppb_ln, mod_As_anom01_B_FU);

**data** GMs_As_bydis; set GM_As_disRA GM_As_disC GM_As_disB GM_As_disO GM_As_anom01_B_FU;

format geomMean LowerCLGeomMean UpperCLGeomMean **8.2**;

**run**;

**data** Ratios_As; set mod_As_disRA mod_As_disC mod_As_disB mod_As_disO mod_As_anom01_B_FU;

Exp_est=exp(estimate);

LL=exp(estimate-**1.96***StdErr);

UL=exp(estimate+**1.96***StdErr);

keep order variable Exp_est LL UL Probt class;

format Exp_est LL UL **8.2**; format Probt **5.3**;

**run**;

%***sort***(work,GMs_As_bydis, order);

%***sort***(work,Ratios_As, order);

**data** Metals_desc_As; merge GMs_As_bydis Ratios_As; by order; **run**;

/*Fe*/

%***ttest*** (labs_FU2, Fe_ppb, disRA, GM_Fe_disRA, **1**, Fe_ppb_ln, mod_Fe_disRA);

%***ttest*** (labs_FU2, Fe_ppb, disC, GM_Fe_disC, **3**, Fe_ppb_ln, mod_Fe_disC);

%***ttest*** (labs_FU2, Fe_ppb, disB, GM_Fe_disB, **4**, Fe_ppb_ln, mod_Fe_disB);

%***ttest*** (labs_FU2, Fe_ppb, disO, GM_Fe_disO, **5**, Fe_ppb_ln, mod_Fe_disO);

%***ttest*** (labs_FU2, Fe_ppb, anom01_B_FU, GM_Fe_anom01_B_FU, **8**, Fe_ppb_ln, mod_Fe_anom01_B_FU);

**data** GMs_Fe_bydis; set GM_Fe_disRA GM_Fe_disC GM_Fe_disB GM_Fe_disO GM_Fe_anom01_B_FU;

format geomMean LowerCLGeomMean UpperCLGeomMean **8.2**;

**run**;

**data** Ratios_Fe; set mod_Fe_disRA mod_Fe_disC mod_Fe_disB mod_Fe_disO mmod_Fe_anom01_B_FU;

Exp_est=exp(estimate);

LL=exp(estimate-**1.96***StdErr);

UL=exp(estimate+**1.96***StdErr);

keep order variable Exp_est LL UL Probt class;

format Exp_est LL UL **8.2**; format Probt **5.3**;

**run**;

%***sort***(work,GMs_Fe_bydis, order);

%***sort***(work,Ratios_Fe, order);

**data** Metals_desc_Fe; merge GMs_Fe_bydis Ratios_Fe; by order; **run**;

/*Mo*/

%***ttest*** (labs_FU2, Mo_ppb, disRA, GM_Mo_disRA, **1**, Mo_ppb_ln, mod_Mo_disRA);

%***ttest*** (labs_FU2, Mo_ppb, disC, GM_Mo_disC, **3**, Mo_ppb_ln, mod_Mo_disC);

%***ttest*** (labs_FU2, Mo_ppb, disB, GM_Mo_disB, **4**, Mo_ppb_ln, mod_Mo_disB);

%***ttest*** (labs_FU2, Mo_ppb, disO, GM_Mo_disO, **5**, Mo_ppb_ln, mod_Mo_disO);

%***ttest*** (labs_FU2, Mo_ppb, anom01_B_FU, GM_Mo_anom01_B_FU, **8**, Mo_ppb_ln, mod_Mo_anom01_B_FU);

**data** GMs_Mo_bydis; set GM_Mo_disRA GM_Mo_disC GM_Mo_disB GM_Mo_disO GM_Mo_anom01_B_FU;

format geomMean LowerCLGeomMean UpperCLGeomMean **8.2**;

**run**;

**data** Ratios_Mo; set mod_Mo_disRA mod_Mo_disC mod_Mo_disB mod_Mo_disO mod_Mo_anom01_B_FU;

Exp_est=exp(estimate);

LL=exp(estimate-**1.96***StdErr);

UL=exp(estimate+**1.96***StdErr);

keep order variable Exp_est LL UL Probt class;

format Exp_est LL UL **8.2**; format Probt **5.3**;

**run**;

%***sort***(work,GMs_Mo_bydis, order);

%***sort***(work,Ratios_Mo, order);

**data** Metals_desc_Mo; merge GMs_Mo_bydis Ratios_Mo; by order; **run**;

/*Mn*/

%***ttest*** (labs_FU2, Mn_ppb, disRA, GM_Mn_disRA, **1**, Mn_ppb_ln, mod_Mn_disRA);

%***ttest*** (labs_FU2, Mn_ppb, disC, GM_Mn_disC, **3**, Mn_ppb_ln, mod_Mn_disC);

%***ttest*** (labs_FU2, Mn_ppb, disB, GM_Mn_disB, **4**, Mn_ppb_ln, mod_Mn_disB);

%***ttest*** (labs_FU2, Mn_ppb, disO, GM_Mn_disO, **5**, Mn_ppb_ln, mod_Mn_disO);

%***ttest*** (labs_FU2, Mn_ppb, anom01_B_FU, GM_Mn_anom01_B_FU, **8**, Mn_ppb_ln, mod_Mn_anom01_B_FU);

**data** GMs_Mn_bydis; set GM_Mn_disRA GM_Mn_disC GM_Mn_disB GM_Mn_disO GM_Mn_anom01_B_FU;

format geomMean LowerCLGeomMean UpperCLGeomMean **8.2**;

**run**;

**data** Ratios_Mn; set mod_Mn_disRA mod_Mn_disC mod_Mn_disB mod_Mn_disO mod_Mn_anom01_B_FU;

Exp_est=exp(estimate);

LL=exp(estimate-**1.96***StdErr);

UL=exp(estimate+**1.96***StdErr);

keep order variable Exp_est LL UL Probt class;

format Exp_est LL UL **8.2**; format Probt **5.3**;

**run**;

%***sort***(work,GMs_Mn_bydis, order);

%***sort***(work,Ratios_Mn, order);

**data** Metals_desc_Mn; merge GMs_Mn_bydis Ratios_Mn; by order; **run**;

/*Ag*/

%***ttest*** (labs_FU2, Ag_ppb, disRA, GM_Ag_disRA, **1**, Ag_ppb_ln, mod_Ag_disRA);

%***ttest*** (labs_FU2, Ag_ppb, disC, GM_Ag_disC, **3**, Ag_ppb_ln, mod_Ag_disC);

%***ttest*** (labs_FU2, Ag_ppb, disB, GM_Ag_disB, **4**, Ag_ppb_ln, mod_Ag_disB);

%***ttest*** (labs_FU2, Ag_ppb, disO, GM_Ag_disO, **5**, Ag_ppb_ln, mod_Ag_disO);

%***ttest*** (labs_FU2, Ag_ppb, anom01_B_FU, GM_Ag_anom01_B_FU, **8**, Ag_ppb_ln, mod_Ag_anom01_B_FU);

**data** GMs_Ag_bydis; set GM_Ag_disRA GM_Ag_disC GM_Ag_disB GM_Ag_disO GM_Ag_anom01_B_FU;

format geomMean LowerCLGeomMean UpperCLGeomMean **8.2**;

**run**;

**data** Ratios_Ag; set mod_Ag_disRA mod_Ag_disC mod_Ag_disB mod_Ag_disO mod_Ag_anom01_B_FU;

Exp_est=exp(estimate);

LL=exp(estimate-**1.96***StdErr);

UL=exp(estimate+**1.96***StdErr);

keep order variable Exp_est LL UL Probt class;

format Exp_est LL UL **8.2**; format Probt **5.3**;

**run**;

%***sort***(work,GMs_Ag_bydis, order);

%***sort***(work,Ratios_Ag, order);

**data** Metals_desc_Ag; merge GMs_Ag_bydis Ratios_Ag; by order; **run**;

/*Pb*/

%***ttest*** (labs_FU2, Pb_ppb, disRA, GM_Pb_disRA, **1**, Pb_ppb_ln, mod_Pb_disRA);

%***ttest*** (labs_FU2, Pb_ppb, disC, GM_Pb_disC, **3**, Pb_ppb_ln, mod_Pb_disC);

%***ttest*** (labs_FU2, Pb_ppb, disB, GM_Pb_disB, **4**, Pb_ppb_ln, mod_Pb_disB);

%***ttest*** (labs_FU2, Pb_ppb, disO, GM_Pb_disO, **5**, Pb_ppb_ln, mod_Pb_disO);

%***ttest*** (labs_FU2, Pb_ppb, anom01_B_FU, GM_Pb_anom01_B_FU, **8**, Pb_ppb_ln, mod_Pb_anom01_B_FU);

**data** GMs_Pb_bydis; set GM_Pb_disRA GM_Pb_disC GM_Pb_disB GM_Pb_disO GM_Pb_anom01_B_FU;

format geomMean LowerCLGeomMean UpperCLGeomMean **8.2**;

**run**;

**data** Ratios_Pb; set mod_Pb_disRA mod_Pb_disC mod_Pb_disB mod_Pb_disO mod_Pb_anom01_B_FU;

Exp_est=exp(estimate);

LL=exp(estimate-**1.96***StdErr);

UL=exp(estimate+**1.96***StdErr);

keep order variable Exp_est LL UL Probt class;

format Exp_est LL UL **8.2**; format Probt **5.3**;

**run**;

%***sort***(work,GMs_Pb_bydis, order);

%***sort***(work,Ratios_Pb, order);

**data** Metals_desc_Pb; merge GMs_Pb_bydis Ratios_Pb; by order; **run**;

/*U*/

%***ttest*** (labs_FU2, U_ppb, disRA, GM_U_disRA, **1**, U_ppb_ln, mod_U_disRA);

%***ttest*** (labs_FU2, U_ppb, disC, GM_U_disC, **3**, U_ppb_ln, mod_U_disC);

%***ttest*** (labs_FU2, U_ppb, disB, GM_U_disB, **4**, U_ppb_ln, mod_U_disB);

%***ttest*** (labs_FU2, U_ppb, disO, GM_U_disO, **5**, U_ppb_ln, mod_U_disO);

%***ttest*** (labs_FU2, U_ppb, anom01_B_FU, GM_U_anom01_B_FU, **8**, U_ppb_ln, mod_U_anom01_B_FU);

**data** GMs_U_bydis; set GM_U_disRA GM_U_disC GM_U_disB GM_U_disO GM_U_anom01_B_FU;

format geomMean LowerCLGeomMean UpperCLGeomMean **8.2**;

**run**;

**data** Ratios_U; set mod_U_disRA mod_U_disC mod_U_disB mod_U_disO mod_U_anom01_B_FU;

Exp_est=exp(estimate);

LL=exp(estimate-**1.96***StdErr);

UL=exp(estimate+**1.96***StdErr);

keep order variable Exp_est LL UL Probt class;

format Exp_est LL UL **8.2**; format Probt **5.3**;

**run**;

%***sort***(work,GMs_U_bydis, order);

%***sort***(work,Ratios_U, order);

**data** Metals_desc_U; merge GMs_U_bydis Ratios_U; by order; **run**;

/*Table 3 - adjust exposure - morbidity links*/;

/* GEE, sandwich estimated errors*/

**%macro** metmodels_adj_GEE (lib, dsn, var1, var2, var3, var4, var5, var6, id);

proc genmod data =&lib.**.**&dsn;

ods output GEEEmpPEst=&var1.vs&var2._GEE;

class &id /param=glm;

model &var1=&var2 &var3 &var4 &var5 &var6/dist=Poisson;

repeated subject=&id;

run;quit;

data &var1._&var2._GEE; set &var1.vs&var2._GEE (keep=Parm Estimate ProbZ); if Parm="&var2."; run;

**%mend** metmodels_adj_GEE;

%***metmodels_adj_GEE***(work, labs_FU2, preterm, Na_r5, age_calc, male, parity_3gr, , code);

%***metmodels_adj_GEE***(work, labs_FU2, preterm, K_r5, age_calc, male, parity_3gr, , code);

%***metmodels_adj_GEE***(work, labs_FU2, preterm, Mg_r5, age_calc, male, parity_3gr, ,code);

%***metmodels_adj_GEE***(work, labs_FU2, preterm, Ca_r5, age_calc, male, parity_3gr, ,code);

%***metmodels_adj_GEE***(work, labs_FU2, preterm, Se_r5, age_calc, male, parity_3gr, ,code);

%***metmodels_adj_GEE***(work, labs_FU2, preterm, Zn_r5, age_calc, male, parity_3gr, ,code);

%***metmodels_adj_GEE***(work, labs_FU2, preterm, Cu_r5, age_calc, male, parity_3gr, ,code);

%***metmodels_adj_GEE***(work, labs_FU2, preterm, Li_r5, age_calc, male, parity_3gr, ,code);

%***metmodels_adj_GEE***(work, labs_FU2, preterm, Co_r5, age_calc, male, parity_3gr, ,code);

%***metmodels_adj_GEE***(work, labs_FU2, preterm, Ni_r5, age_calc, male, parity_3gr, ,code);

%***metmodels_adj_GEE***(work, labs_FU2, preterm, Tl_r5, age_calc, male, parity_3gr, ,code);

%***metmodels_adj_GEE***(work, labs_FU2, preterm, Al_r5, age_calc, male, parity_3gr, ,code);

%***metmodels_adj_GEE***(work, labs_FU2, preterm, Cr_r5, age_calc, male, parity_3gr, ,code);

%***metmodels_adj_GEE***(work, labs_FU2, preterm, Sr_r5, age_calc, male, parity_3gr, ,code);

%***metmodels_adj_GEE***(work, labs_FU2, preterm, Ba_r5, age_calc, male, parity_3gr, ,code);

%***metmodels_adj_GEE***(work, labs_FU2, preterm, Cd_r5, age_calc, male, parity_3gr, ,code);

%***metmodels_adj_GEE***(work, labs_FU2, preterm, Be_r5, age_calc, male, parity_3gr, ,code);

%***metmodels_adj_GEE***(work, labs_FU2, preterm, V_r5, age_calc, male, parity_3gr, ,code);

%***metmodels_adj_GEE***(work, labs_FU2, preterm, As_r5, age_calc, male, parity_3gr, ,code);

%***metmodels_adj_GEE***(work, labs_FU2, preterm, Fe_r5, age_calc, male, parity_3gr, ,code);

%***metmodels_adj_GEE***(work, labs_FU2, preterm, Mo_r5, age_calc, male, parity_3gr, ,code);

%***metmodels_adj_GEE***(work, labs_FU2, preterm, Mn_r5, age_calc, male, parity_3gr, ,code);

%***metmodels_adj_GEE***(work, labs_FU2, preterm, Ag_r5, age_calc, male, parity_3gr, ,code);

%***metmodels_adj_GEE***(work, labs_FU2, preterm, Pb_r5, age_calc, male, parity_3gr, ,code);

%***metmodels_adj_GEE***(work, labs_FU2, preterm, U_r5, age_calc, male, parity_3gr, ,code);

**data** models_preterm_r5_GEE; set Preterm_na_r5_GEE Preterm_k_r5_GEE Preterm_mg_r5_GEE Preterm_ca_r5_GEE Preterm_se_r5_GEE

Preterm_zn_r5_GEE Preterm_cu_r5_GEE Preterm_li_r5_GEE Preterm_co_r5_GEE Preterm_ni_r5_GEE

Preterm_tl_r5_GEE Preterm_al_r5_GEE Preterm_cr_r5_GEE Preterm_sr_r5_GEE Preterm_ba_r5_GEE

Preterm_cd_r5_GEE Preterm_be_r5_GEE Preterm_v_r5_GEE Preterm_as_r5_GEE Preterm_fe_r5_GEE

Preterm_mo_r5_GEE Preterm_mn_r5_GEE Preterm_ag_r5_GEE Preterm_pb_r5_GEE Preterm_u_r5_GEE;

**run**;

%***metmodels_adj_GEE***(work, labs_FU2, disRA, Na_r5, parity_3gr, age_calc, male, preterm, code);

%***metmodels_adj_GEE***(work, labs_FU2, disRA, K_r5, parity_3gr, age_calc, male, preterm, code);

%***metmodels_adj_GEE***(work, labs_FU2, disRA, Mg_r5, parity_3gr, age_calc, male, preterm, code);

%***metmodels_adj_GEE***(work, labs_FU2, disRA, Ca_r5, parity_3gr, age_calc, male, preterm, code);

%***metmodels_adj_GEE***(work, labs_FU2, disRA, Se_r5, parity_3gr, age_calc, male, preterm, code);

%***metmodels_adj_GEE***(work, labs_FU2, disRA, Zn_r5, parity_3gr, age_calc, male, preterm, code);

%***metmodels_adj_GEE***(work, labs_FU2, disRA, Cu_r5, parity_3gr, age_calc, male, preterm, codee);

%***metmodels_adj_GEE***(work, labs_FU2, disRA, Li_r5, parity_3gr, age_calc, male, preterm, code);

%***metmodels_adj_GEE***(work, labs_FU2, disRA, Co_r5, parity_3gr, age_calc, male, preterm, code);

%***metmodels_adj_GEE***(work, labs_FU2, disRA, Ni_r5, parity_3gr, age_calc, male, preterm, codee);

%***metmodels_adj_GEE***(work, labs_FU2, disRA, Tl_r5, parity_3gr, age_calc, male, preterm, code);

%***metmodels_adj_GEE***(work, labs_FU2, disRA, Al_r5, parity_3gr, age_calc, male, preterm, code);

%***metmodels_adj_GEE***(work, labs_FU2, disRA, Cr_r5, parity_3gr, age_calc, male, preterm, code);

%***metmodels_adj_GEE***(work, labs_FU2, disRA, Sr_r5, parity_3gr,age_calc, male, preterm, code);

%***metmodels_adj_GEE***(work, labs_FU2, disRA, Ba_r5, parity_3gr,age_calc, male, preterm, code);

%***metmodels_adj_GEE***(work, labs_FU2, disRA, Cd_r5, parity_3gr,age_calc, male, preterm, code);

%***metmodels_adj_GEE***(work, labs_FU2, disRA, Be_r5, parity_3gr,age_calc, male, preterm, code);

%***metmodels_adj_GEE***(work, labs_FU2, disRA, V_r5, parity_3gr,age_calc, male, preterm, codee);

%***metmodels_adj_GEE***(work, labs_FU2, disRA, As_r5, parity_3gr,age_calc, male, preterm, code);

%***metmodels_adj_GEE***(work, labs_FU2, disRA, Fe_r5, parity_3gr,age_calc, male, preterm, code);

%***metmodels_adj_GEE***(work, labs_FU2, disRA, Mo_r5, parity_3gr,age_calc, male, preterm, code);

%***metmodels_adj_GEE***(work, labs_FU2, disRA, Mn_r5, parity_3gr,age_calc, male, preterm, code);

%***metmodels_adj_GEE***(work, labs_FU2, disRA, Ag_r5, parity_3gr,age_calc, male, preterm, code);

%***metmodels_adj_GEE***(work, labs_FU2, disRA, Pb_r5, parity_3gr,age_calc, male, preterm, code);

%***metmodels_adj_GEE***(work, labs_FU2, disRA, U_r5, parity_3gr,age_calc, male, preterm, code);

**data** models_disRA_r5_GEE; set DisRA_na_r5_GEE DisRA_k_r5_GEE DisRA_mg_r5_GEE DisRA_ca_r5_GEE DisRA_se_r5_GEE

DisRA_zn_r5_GEE DisRA_cu_r5_GEE DisRA_li_r5_GEE DisRA_co_r5_GEE DisRA_ni_r5_GEE

DisRA_tl_r5_GEE DisRA_al_r5_GEE DisRA_cr_r5_GEE DisRA_sr_r5_GEE DisRA_ba_r5_GEE

DisRA_cd_r5_GEE DisRA_be_r5_GEE DisRA_v_r5_GEE DisRA_as_r5_GEE DisRA_fe_r5_GEE

DisRA_mo_r5_GEE DisRA_mn_r5_GEE DisRA_ag_r5_GEE DisRA_pb_r5_GEE DisRA_u_r5_GEE;

**run**;

%***metmodels_adj_GEE***(work, labs_FU2, disC, Na_r5, parity_3gr, age_calc, male, preterm,code);

%***metmodels_adj_GEE***(work, labs_FU2, disC, K_r5, parity_3gr, age_calc, male,preterm ,code);

%***metmodels_adj_GEE***(work, labs_FU2, disC, Mg_r5, parity_3gr, age_calc, male,preterm ,code);

%***metmodels_adj_GEE***(work, labs_FU2, disC, Ca_r5, parity_3gr, age_calc, male, preterm,code);

%***metmodels_adj_GEE***(work, labs_FU2, disC, Se_r5, parity_3gr, age_calc, male, preterm,code);

%***metmodels_adj_GEE***(work, labs_FU2, disC, Zn_r5, parity_3gr, age_calc, male, preterm,code);

%***metmodels_adj_GEE***(work, labs_FU2, disC, Cu_r5, parity_3gr, age_calc, male, preterm,code);

%***metmodels_adj_GEE***(work, labs_FU2, disC, Li_r5, parity_3gr, age_calc, male,preterm,code);

%***metmodels_adj_GEE***(work, labs_FU2, disC, Co_r5, parity_3gr, age_calc, male,preterm ,code);

%***metmodels_adj_GEE***(work, labs_FU2, disC, Ni_r5, parity_3gr, age_calc, male, preterm,code);

%***metmodels_adj_GEE***(work, labs_FU2, disC, Tl_r5, parity_3gr, age_calc, male, preterm,code);

%***metmodels_adj_GEE***(work, labs_FU2, disC, Al_r5, parity_3gr, age_calc, male,preterm ,code);

%***metmodels_adj_GEE***(work, labs_FU2, disC, Cr_r5, parity_3gr, age_calc, male, preterm,code);

%***metmodels_adj_GEE***(work, labs_FU2, disC, Sr_r5, parity_3gr, age_calc, male,preterm ,code);

%***metmodels_adj_GEE***(work, labs_FU2, disC, Ba_r5, parity_3gr, age_calc, male, preterm,code);

%***metmodels_adj_GEE***(work, labs_FU2, disC, Cd_r5, parity_3gr, age_calc, male,preterm ,code);

%***metmodels_adj_GEE***(work, labs_FU2, disC, Be_r5, parity_3gr, age_calc, male, preterm,code);

%***metmodels_adj_GEE***(work, labs_FU2, disC, V_r5, parity_3gr, age_calc, male, preterm,code);

%***metmodels_adj_GEE***(work, labs_FU2, disC, As_r5, parity_3gr, age_calc, male,preterm ,code);

%***metmodels_adj_GEE***(work, labs_FU2, disC, Fe_r5, parity_3gr, age_calc, male,preterm ,code);

%***metmodels_adj_GEE***(work, labs_FU2, disC, Mo_r5, parity_3gr, age_calc, male, preterm,code);

%***metmodels_adj_GEE***(work, labs_FU2, disC, Mn_r5, parity_3gr, age_calc, male,preterm ,code);

%***metmodels_adj_GEE***(work, labs_FU2, disC, Ag_r5, parity_3gr, age_calc, male, preterm,code);

%***metmodels_adj_GEE***(work, labs_FU2, disC, Pb_r5, parity_3gr, age_calc, male,preterm ,code);

%***metmodels_adj_GEE***(work, labs_FU2, disC, U_r5, parity_3gr, age_calc, male, preterm,code);

**data** models_disC_r5_GEE; set DisC_na_r5_GEE DisC_k_r5_GEE DisC_mg_r5_GEE DisC_ca_r5_GEE DisC_se_r5_GEE

DisC_zn_r5_GEE DisC_cu_r5_GEE DisC_li_r5_GEE DisC_co_r5_GEE DisC_ni_r5_GEE

DisC_tl_r5_GEE DisC_al_r5_GEE DisC_cr_r5_GEE DisC_sr_r5_GEE DisC_ba_r5_GEE

DisC_cd_r5_GEE DisC_be_r5_GEE DisC_v_r5_GEE DisC_as_r5_GEE DisC_fe_r5_GEE

DisC_mo_r5_GEE DisC_mn_r5_GEE DisC_ag_r5_GEE DisC_pb_r5_GEE DisC_u_r5_GEE;

**run**;

%***metmodels_adj_GEE***(work, labs_FU2, disB, Na_r5, parity_3gr, male, preterm, age_calc,code);

%***metmodels_adj_GEE***(work, labs_FU2, disB, K_r5, parity_3gr, male, preterm, age_calc,code);

%***metmodels_adj_GEE***(work, labs_FU2, disB, Mg_r5, parity_3gr, male, preterm,age_calc ,code);

%***metmodels_adj_GEE***(work, labs_FU2, disB, Ca_r5, parity_3gr, male, preterm,age_calc ,code);

%***metmodels_adj_GEE***(work, labs_FU2, disB, Se_r5, parity_3gr, male, preterm, age_calc,code);

%***metmodels_adj_GEE***(work, labs_FU2, disB, Zn_r5, parity_3gr, male, preterm, age_calc,code);

%***metmodels_adj_GEE***(work, labs_FU2, disB, Cu_r5, parity_3gr, male, preterm, age_calc,code);

%***metmodels_adj_GEE***(work, labs_FU2, disB, Li_r5, parity_3gr, male, preterm, age_calc,code);

%***metmodels_adj_GEE***(work, labs_FU2, disB, Co_r5, parity_3gr, male, preterm,age_calc ,code);

%***metmodels_adj_GEE***(work, labs_FU2, disB, Ni_r5, parity_3gr, male, preterm, age_calc,code);

%***metmodels_adj_GEE***(work, labs_FU2, disB, Tl_r5, parity_3gr, male, preterm, age_calc,code);

%***metmodels_adj_GEE***(work, labs_FU2, disB, Al_r5, parity_3gr, male, preterm, age_calc,code);

%***metmodels_adj_GEE***(work, labs_FU2, disB, Cr_r5, parity_3gr, male, preterm,age_calc ,code);

%***metmodels_adj_GEE***(work, labs_FU2, disB, Sr_r5, parity_3gr, male, preterm,age_calc ,code);

%***metmodels_adj_GEE***(work, labs_FU2, disB, Ba_r5, parity_3gr, male, preterm,age_calc,code);

%***metmodels_adj_GEE***(work, labs_FU2, disB, Cd_r5, parity_3gr, male, preterm, age_calc,code);

%***metmodels_adj_GEE***(work, labs_FU2, disB, Be_r5, parity_3gr, male, preterm,age_calc ,code);

%***metmodels_adj_GEE***(work, labs_FU2, disB, V_r5, parity_3gr, male, preterm, age_calc,code);

%***metmodels_adj_GEE***(work, labs_FU2, disB, As_r5, parity_3gr, male, preterm, age_calc,code);

%***metmodels_adj_GEE***(work, labs_FU2, disB, Fe_r5, parity_3gr, male, preterm,age_calc,code);

%***metmodels_adj_GEE***(work, labs_FU2, disB, Mo_r5, parity_3gr, male, preterm, age_calc,code);

%***metmodels_adj_GEE***(work, labs_FU2, disB, Mn_r5, parity_3gr, male, preterm,age_calc ,code);

%***metmodels_adj_GEE***(work, labs_FU2, disB, Ag_r5, parity_3gr, male, preterm,age_calc ,code);

%***metmodels_adj_GEE***(work, labs_FU2, disB, Pb_r5, parity_3gr, male, preterm,age_calc ,code);

%***metmodels_adj_GEE***(work, labs_FU2, disB, U_r5, parity_3gr, male, preterm,age_calc ,code);

**data** models_disB_r5_GEE; set DisB_na_r5_GEE DisB_k_r5_GEE DisB_mg_r5_GEE DisB_ca_r5_GEE DisB_se_r5_GEE

DisB_zn_r5_GEE DisB_cu_r5_GEE DisB_li_r5_GEE DisB_co_r5_GEE DisB_ni_r5_GEE

DisB_tl_r5_GEE DisB_al_r5_GEE DisB_cr_r5_GEE DisB_sr_r5_GEE DisB_ba_r5_GEE

DisB_cd_r5_GEE DisB_be_r5_GEE DisB_v_r5_GEE DisB_as_r5_GEE DisB_fe_r5_GEE

DisB_mo_r5_GEE DisB_mn_r5_GEE DisB_ag_r5_GEE DisB_pb_r5_GEE DisB_u_r5_GEE;

**run**;

%***metmodels_adj_GEE***(work, labs_FU2, disO, Na_r5, parity_3gr , preterm, male, age_calc, code);

%***metmodels_adj_GEE***(work, labs_FU2, disO, K_r5, parity_3gr , preterm, male, age_calc , code);

%***metmodels_adj_GEE***(work, labs_FU2, disO, Mg_r5, parity_3gr , preterm, male, age_calc , code);

%***metmodels_adj_GEE***(work, labs_FU2, disO, Ca_r5, parity_3gr , preterm, male, age_calc , code);

%***metmodels_adj_GEE***(work, labs_FU2, disO, Se_r5, parity_3gr , preterm, male, age_calc , code);

%***metmodels_adj_GEE***(work, labs_FU2, disO, Zn_r5, parity_3gr , preterm,male, age_calc , code);

%***metmodels_adj_GEE***(work, labs_FU2, disO, Cu_r5, parity_3gr , preterm, male, age_calc , code);

%***metmodels_adj_GEE***(work, labs_FU2, disO, Li_r5, parity_3gr , preterm, male, age_calc , code);

%***metmodels_adj_GEE***(work, labs_FU2, disO, Co_r5, parity_3gr , preterm, male, age_calc , code);

%***metmodels_adj_GEE***(work, labs_FU2, disO, Ni_r5, parity_3gr , preterm, male, age_calc , code);

%***metmodels_adj_GEE***(work, labs_FU2, disO, Tl_r5, parity_3gr , preterm, male, age_calc , code);

%***metmodels_adj_GEE***(work, labs_FU2, disO, Al_r5, parity_3gr , preterm, male, age_calc , code);

%***metmodels_adj_GEE***(work, labs_FU2, disO, Cr_r5, parity_3gr , preterm, male, age_calc , code);

%***metmodels_adj_GEE***(work, labs_FU2, disO, Sr_r5, parity_3gr , preterm, male, age_calc , code);

%***metmodels_adj_GEE***(work, labs_FU2, disO, Ba_r5, parity_3gr , preterm, male, age_calc , code);

%***metmodels_adj_GEE***(work, labs_FU2, disO, Cd_r5, parity_3gr , preterm, male, age_calc , code);

%***metmodels_adj_GEE***(work, labs_FU2, disO, Be_r5, parity_3gr , preterm, male, age_calc , code);

%***metmodels_adj_GEE***(work, labs_FU2, disO, V_r5, parity_3gr , preterm,male, age_calc , code);

%***metmodels_adj_GEE***(work, labs_FU2, disO, As_r5, parity_3gr , preterm, male, age_calc , code);

%***metmodels_adj_GEE***(work, labs_FU2, disO, Fe_r5, parity_3gr , preterm, male, age_calc , code);

%***metmodels_adj_GEE***(work, labs_FU2, disO, Mo_r5, parity_3gr , preterm, male, age_calc , code);

%***metmodels_adj_GEE***(work, labs_FU2, disO, Mn_r5, parity_3gr , preterm, male, age_calc , code);

%***metmodels_adj_GEE***(work, labs_FU2, disO, Ag_r5, parity_3gr , preterm, male, age_calc , code);

%***metmodels_adj_GEE***(work, labs_FU2, disO, Pb_r5, parity_3gr , preterm, male, age_calc , code);

%***metmodels_adj_GEE***(work, labs_FU2, disO, U_r5, parity_3gr , preterm, male, age_calc, code);

**data** models_disO_r5_GEE; set disO_na_r5_GEE disO_k_r5_GEE disO_mg_r5_GEE disO_ca_r5_GEE disO_se_r5_GEE

disO_zn_r5_GEE disO_cu_r5_GEE disO_li_r5_GEE disO_co_r5_GEE disO_ni_r5_GEE

disO_tl_r5_GEE disO_al_r5_GEE disO_cr_r5_GEE disO_sr_r5_GEE disO_ba_r5_GEE

disO_cd_r5_GEE disO_be_r5_GEE disO_v_r5_GEE disO_as_r5_GEE disO_fe_r5_GEE

disO_mo_r5_GEE disO_mn_r5_GEE disO_ag_r5_GEE disO_pb_r5_GEE disO_u_r5_GEE;

**run**;

%***metmodels_adj_GEE***(work, labs_FU2, anom01_B_FU, Na_r5, male, preterm,age_calc ,parity_3gr , code);

%***metmodels_adj_GEE***(work, labs_FU2, anom01_B_FU, K_r5, male, preterm,age_calc ,parity_3gr , code);

%***metmodels_adj_GEE***(work, labs_FU2, anom01_B_FU, Mg_r5, male, preterm,age_calc ,parity_3gr, code);

%***metmodels_adj_GEE***(work, labs_FU2, anom01_B_FU, Ca_r5, male, preterm,age_calc ,parity_3gr, code);

%***metmodels_adj_GEE***(work, labs_FU2, anom01_B_FU, Se_r5, male, preterm,age_calc ,parity_3gr , code);

%***metmodels_adj_GEE***(work, labs_FU2, anom01_B_FU, Zn_r5, male, preterm,age_calc ,parity_3gr , code);

%***metmodels_adj_GEE***(work, labs_FU2, anom01_B_FU, Cu_r5, male, preterm,age_calc ,parity_3gr , code);

%***metmodels_adj_GEE***(work, labs_FU2, anom01_B_FU, Li_r5, male, preterm,age_calc ,parity_3gr , code);

%***metmodels_adj_GEE***(work, labs_FU2, anom01_B_FU, Co_r5, male, preterm,age_calc ,parity_3gr , code);

%***metmodels_adj_GEE***(work, labs_FU2, anom01_B_FU, Ni_r5, male,preterm,age_calc ,parity_3gr , code);

%***metmodels_adj_GEE***(work, labs_FU2, anom01_B_FU, Tl_r5, male, preterm,age_calc ,parity_3gr , code);

%***metmodels_adj_GEE***(work, labs_FU2, anom01_B_FU, Al_r5, male, preterm,age_calc ,parity_3gr , code);

%***metmodels_adj_GEE***(work, labs_FU2, anom01_B_FU, Cr_r5, male, preterm,age_calc ,parity_3gr , code);

%***metmodels_adj_GEE***(work, labs_FU2, anom01_B_FU, Sr_r5, male, preterm,age_calc ,parity_3gr, code);

%***metmodels_adj_GEE***(work, labs_FU2, anom01_B_FU, Ba_r5, male, preterm,age_calc ,parity_3gr, code);

%***metmodels_adj_GEE***(work, labs_FU2, anom01_B_FU, Cd_r5, male,preterm,age_calc ,parity_3gr , code);

%***metmodels_adj_GEE***(work, labs_FU2, anom01_B_FU, Be_r5, male, preterm,age_calc ,parity_3gr, code);

%***metmodels_adj_GEE***(work, labs_FU2, anom01_B_FU, V_r5, male, preterm,age_calc ,parity_3gr , code);

%***metmodels_adj_GEE***(work, labs_FU2, anom01_B_FU, As_r5, male, preterm,age_calc ,parity_3gr , code);

%***metmodels_adj_GEE***(work, labs_FU2, anom01_B_FU, Fe_r5, male, preterm,age_calc ,parity_3gr,code);

%***metmodels_adj_GEE***(work, labs_FU2, anom01_B_FU, Mo_r5, male, preterm,age_calc ,parity_3gr , code);

%***metmodels_adj_GEE***(work, labs_FU2, anom01_B_FU, Mn_r5, male, preterm,age_calc ,parity_3gr , code);

%***metmodels_adj_GEE***(work, labs_FU2, anom01_B_FU, Ag_r5, male,preterm,age_calc ,parity_3gr, code);

%***metmodels_adj_GEE***(work, labs_FU2, anom01_B_FU, Pb_r5, male, preterm,age_calc ,parity_3gr , code);

%***metmodels_adj_GEE***(work, labs_FU2, anom01_B_FU, U_r5, male, preterm,age_calc ,parity_3gr , code);

**data** models_anom01_B_FU_r5_GEE; set anom01_B_FU_na_r5_GEE anom01_B_FU_k_r5_GEE anom01_B_FU_mg_r5_GEE anom01_B_FU_ca_r5_GEE anom01_B_FU_se_r5_GEE

anom01_B_FU_zn_r5_GEE anom01_B_FU_cu_r5_GEE anom01_B_FU_li_r5_GEE anom01_B_FU_co_r5_GEE anom01_B_FU_ni_r5_GEE

anom01_B_FU_tl_r5_GEE anom01_B_FU_al_r5_GEE anom01_B_FU_cr_r5_GEE anom01_B_FU_sr_r5_GEE anom01_B_FU_ba_r5_GEE

anom01_B_FU_cd_r5_GEE anom01_B_FU_be_r5_GEE anom01_B_FU_v_r5_GEE anom01_B_FU_as_r5_GEE anom01_B_FU_fe_r5_GEE

anom01_B_FU_mo_r5_GEE anom01_B_FU_mn_r5_GEE anom01_B_FU_ag_r5_GEE anom01_B_FU_pb_r5_GEE anom01_B_FU_u_r5_GEE;

**run**;

%***sort***(work,models_preterm_r5_GEE , Parm);

%***sort***(work,models_disRA_r5_GEE , Parm);

%***sort***(work,models_disC_r5_GEE , Parm);

%***sort***(work,models_disB_r5_GEE , Parm);

%***sort***(work,models_disO_r5_GEE , Parm);

%***sort***(work,models_anom01_B_FU_r5_GEE , Parm);

**data** models_r5_GEE; merge models_preterm_r5_GEE (rename=(Estimate=Est_preterm ProbZ=pv_preterm))

models_disRA_r5_GEE (rename=(Estimate=Est_disRA ProbZ=pv_disRA))

models_disC_r5_GEE (rename=(Estimate=Est_disC ProbZ=pv_disC))

models_disB_r5_GEE (rename=(Estimate=Est_disB ProbZ=pv_disB))

models_disO_r5_GEE (rename=(Estimate=Est_disO ProbZ=pv_disO))

models_anom01_B_FU_r5_GEE (rename=(Estimate=Est_anom ProbZ=pv_anom));

by Parm;

**run**;

/*******Histograms of metals by morbidity status*/

**data** data_graph; set data (keep=code preterm disRA disC disB disO anom01_B_FU disS

LI_ppb Be_ppb Na_ppm Mg_ppm Al_ppb K_ppm Ca_ppm V_ppb Cr_ppb Mn_ppb Fe_ppb Co_ppb Ni_ppb Cu_ppb Zn_ppb

As_ppb Se_ppb Sr_ppb Mo_ppb Ag_ppb Cd_ppb

Ba_ppb Tl_ppb Pb_ppb U_ppb

LI_ppb_ln Be_ppb_ln Na_ppm_ln Mg_ppm_ln Al_ppb_ln K_ppm_ln Ca_ppm_ln V_ppb_ln Cr_ppb_ln Mn_ppb_ln Fe_ppb_ln Co_ppb_ln Ni_ppb_ln Cu_ppb_ln Zn_ppb_ln

As_ppb_ln Se_ppb_ln Sr_ppb_ln Mo_ppb_ln Ag_ppb_ln Cd_ppb_ln

Ba_ppb_ln Tl_ppb_ln Pb_ppb_ln U_ppb_ln);

**run**;

/*an example for preterm*/

**proc** **sgplot** data=data_graph NOAUTOLEGEND;

histogram Ca_ppm/ GROUP=preterm binstart=**42** binwidth=**3** transparency=**0.5**;

xaxis label="Ca, ppm" labelattrs=(size=**24**pt weight=bold family='Ariel');

yaxis label="Frequency, %" labelattrs=(size=**11**pt weight=bold family='Times New Roman') values=(**0** to **100** by **10**);

density Ca_ppm / type=kernel GROUP=preterm;

**run**;

**proc** **sgplot** data=data_graph NOAUTOLEGEND;

histogram Cu_ppb/ GROUP=preterm binstart=**42** binwidth=**3** transparency=**0.5**;

xaxis label="Cu, ppb" labelattrs=(size=**24**pt weight=bold family='Ariel');

yaxis label="Frequency, %" labelattrs=(size=**11**pt weight=bold family='Times New Roman') values=(**0** to **100** by **10**);

density Cu_ppb / type=kernel GROUP=preterm;

**run**;

**proc** **sgplot** data=data_graph NOAUTOLEGEND;

histogram K_ppm/ GROUP=preterm binstart=**42** binwidth=**3** transparency=**0.5**;

xaxis label="K, ppm" labelattrs=(size=**24**pt weight=bold family='Ariel');

yaxis label="Frequency, %" labelattrs=(size=**11**pt weight=bold family='Times New Roman') values=(**0** to **100** by **10**);

density K_ppm / type=kernel GROUP=preterm;

**run**;

**proc** **sgplot** data=data_graph NOAUTOLEGEND;

histogram Mg_ppm/ GROUP=preterm binstart=**42** binwidth=**3** transparency=**0.5**;

xaxis label="Mg, ppm" labelattrs=(size=**24**pt weight=bold family='Ariel');

yaxis label="Frequency, %" labelattrs=(size=**11**pt weight=bold family='Times New Roman') values=(**0** to **100** by **10**);

density Mg_ppm / type=kernel GROUP=preterm;

**run**;

**proc** **sgplot** data=data_graph NOAUTOLEGEND;

histogram Na_ppm/ GROUP=preterm binstart=**42** binwidth=**3** transparency=**0.5**;

xaxis label="Na, ppm" labelattrs=(size=**24**pt weight=bold family='Ariel');

yaxis label="Frequency, %" labelattrs=(size=**11**pt weight=bold family='Times New Roman' ) values=(**0** to **100** by **10**);

density Na_ppm / type=kernel GROUP=preterm;

**run**;

**proc** **sgplot** data=data_graph NOAUTOLEGEND;

histogram Se_ppb/ GROUP=preterm binstart=**42** binwidth=**3** transparency=**0.5**;

xaxis label="Se, ppb" labelattrs=(size=**24**pt weight=bold family='Ariel');

yaxis label="Frequency, %" labelattrs=(size=**11**pt weight=bold family='Times New Roman') values=(**0** to **100** by **10**);

density Se_ppb / type=kernel GROUP=preterm;

**run**;

**proc** **sgplot** data=data_graph NOAUTOLEGEND;

histogram Zn_ppb/ GROUP=preterm binstart=**42** binwidth=**3** transparency=**0.5**;

xaxis label="Zn, ppb" labelattrs=(size=**24**pt weight=bold family='Ariel');

yaxis label="Frequency, %" labelattrs=(size=**11**pt weight=bold family='Times New Roman')values=(**0** to **100** by **10**);

density Zn_ppb / type=kernel GROUP=preterm;

**run**;

**proc** **sgplot** data=data_graph NOAUTOLEGEND;

histogram Ag_ppb/ GROUP=preterm binstart=**42** binwidth=**3** transparency=**0.5**;

xaxis label="Ag, ppb" labelattrs=(size=**24**pt weight=bold family='Ariel');

yaxis label="Frequency, %" labelattrs=(size=**11**pt weight=bold family='Times New Roman')values=(**0** to **100** by **10**);

density Ag_ppb / type=kernel GROUP=preterm;

**run**;

**proc** **sgplot** data=data_graph NOAUTOLEGEND;

histogram Al_ppb/ GROUP=preterm binstart=**42** binwidth=**3** transparency=**0.5**;

xaxis label="Al, ppb" labelattrs=(size=**24**pt weight=bold family='Ariel');

yaxis label="Frequency, %" labelattrs=(size=**11**pt weight=bold family='Times New Roman')values=(**0** to **100** by **10**);

density Al_ppb / type=kernel GROUP=preterm;

**run**;

**proc** **sgplot** data=data_graph NOAUTOLEGEND;

histogram As_ppb/ GROUP=preterm binstart=**42** binwidth=**3** transparency=**0.5**;

xaxis label="As, ppb" labelattrs=(size=**24**pt weight=bold family='Ariel');

yaxis label="Frequency, %" labelattrs=(size=**11**pt weight=bold family='Times New Roman')values=(**0** to **100** by **10**);

density As_ppb / type=kernel GROUP=preterm;

**run**;

**proc** **sgplot** data=data_graph NOAUTOLEGEND;

histogram Ba_ppb/ GROUP=preterm binstart=**42** binwidth=**3** transparency=**0.5**;

xaxis label="Ba, ppb" labelattrs=(size=**24**pt weight=bold family='Ariel');

yaxis label="Frequency, %" labelattrs=(size=**11**pt weight=bold family='Times New Roman')values=(**0** to **100** by **10**);

density Ba_ppb / type=kernel GROUP=preterm;

**run**;

**proc** **sgplot** data=data_graph NOAUTOLEGEND;

histogram Be_ppb/ GROUP=preterm binstart=**42** binwidth=**3** transparency=**0.5**;

xaxis label="Be, ppb" labelattrs=(size=**24**pt weight=bold family='Ariel');

yaxis label="Frequency, %" labelattrs=(size=**11**pt weight=bold family='Times New Roman')values=(**0** to **100** by **10**);

density Be_ppb / type=kernel GROUP=preterm;

**run**;

**proc** **sgplot** data=data_graph NOAUTOLEGEND;

histogram Cd_ppb/ GROUP=preterm binstart=**42** binwidth=**3** transparency=**0.5**;

xaxis label="Cd, ppb" labelattrs=(size=**24**pt weight=bold family='Ariel');

yaxis label="Frequency, %" labelattrs=(size=**11**pt weight=bold family='Times New Roman')values=(**0** to **100** by **10**);

density Cd_ppb / type=kernel GROUP=preterm;

**run**;

**proc** **sgplot** data=data_graph NOAUTOLEGEND;

histogram Co_ppb/ GROUP=preterm binstart=**42** binwidth=**3** transparency=**0.5**;

xaxis label="Co, ppb" labelattrs=(size=**24**pt weight=bold family='Ariel');

yaxis label="Frequency, %" labelattrs=(size=**11**pt weight=bold family='Times New Roman')values=(**0** to **100** by **10**);

density Co_ppb / type=kernel GROUP=preterm;

**run**;

**proc** **sgplot** data=data_graph NOAUTOLEGEND;

histogram Cr_ppb/ GROUP=preterm binstart=**42** binwidth=**3** transparency=**0.5**;

xaxis label="Cr, ppb" labelattrs=(size=**24**pt weight=bold family='Ariel');

yaxis label="Frequency, %" labelattrs=(size=**11**pt weight=bold family='Times New Roman')values=(**0** to **100** by **10**);

density Cr_ppb / type=kernel GROUP=preterm;

**run**;

**proc** **sgplot** data=data_graph NOAUTOLEGEND;

histogram Fe_ppb/ GROUP=preterm binstart=**42** binwidth=**3** transparency=**0.5**;

xaxis label="Fe, ppb" labelattrs=(size=**24**pt weight=bold family='Ariel');

yaxis label="Frequency, %" labelattrs=(size=**11**pt weight=bold family='Times New Roman')values=(**0** to **100** by **10**);

density Fe_ppb / type=kernel GROUP=preterm;

**run**;

**proc** **sgplot** data=data_graph NOAUTOLEGEND;

histogram Li_ppb/ GROUP=preterm binstart=**42** binwidth=**3** transparency=**0.5**;

xaxis label="Li, ppb" labelattrs=(size=**24**pt weight=bold family='Ariel');

yaxis label="Frequency, %" labelattrs=(size=**11**pt weight=bold family='Times New Roman')values=(**0** to **100** by **10**);

density Li_ppb / type=kernel GROUP=preterm;

**run**;

**proc** **sgplot** data=data_graph NOAUTOLEGEND;

histogram Mn_ppb/ GROUP=preterm binstart=**42** binwidth=**3** transparency=**0.5**;

xaxis label="Mn, ppb" labelattrs=(size=**24**pt weight=bold family='Ariel');

yaxis label="Frequency, %" labelattrs=(size=**11**pt weight=bold family='Times New Roman')values=(**0** to **100** by **10**);

density Mn_ppb / type=kernel GROUP=preterm;

**run**;

**proc** **sgplot** data=data_graph NOAUTOLEGEND;

histogram Mo_ppb/ GROUP=preterm binstart=**42** binwidth=**3** transparency=**0.5**;

xaxis label="Mo, ppb" labelattrs=(size=**24**pt weight=bold family='Ariel');

yaxis label="Frequency, %" labelattrs=(size=**11**pt weight=bold family='Times New Roman')values=(**0** to **100** by **10**);

density Mo_ppb / type=kernel GROUP=preterm;

**run**;

**proc** **sgplot** data=data_graph NOAUTOLEGEND;

histogram Ni_ppb/ GROUP=preterm binstart=**42** binwidth=**3** transparency=**0.5**;

xaxis label="Ni, ppb" labelattrs=(size=**24**pt weight=bold family='Ariel');

yaxis label="Frequency, %" labelattrs=(size=**11**pt weight=bold family='Times New Roman')values=(**0** to **100** by **10**);

density Ni_ppb / type=kernel GROUP=preterm;

**run**;

**proc** **sgplot** data=data_graph NOAUTOLEGEND;

histogram Pb_ppb/ GROUP=preterm binstart=**42** binwidth=**3** transparency=**0.5**;

xaxis label="Pb, ppb" labelattrs=(size=**24**pt weight=bold family='Ariel');

yaxis label="Frequency, %" labelattrs=(size=**11**pt weight=bold family='Times New Roman')values=(**0** to **100** by **10**);

density Pb_ppb / type=kernel GROUP=preterm;

**run**;

**proc** **sgplot** data=data_graph NOAUTOLEGEND;

histogram Sr_ppb/ GROUP=preterm binstart=**42** binwidth=**3** transparency=**0.5**;

xaxis label="Sr, ppb" labelattrs=(size=**24**pt weight=bold family='Ariel');

yaxis label="Frequency, %" labelattrs=(size=**11**pt weight=bold family='Times New Roman')values=(**0** to **100** by **10**);

density Sr_ppb / type=kernel GROUP=preterm;

**run**;

**proc** **sgplot** data=data_graph NOAUTOLEGEND;

histogram Tl_ppb/ GROUP=preterm binstart=**42** binwidth=**3** transparency=**0.5**;

xaxis label="Tl, ppb" labelattrs=(size=**24**pt weight=bold family='Ariel');

yaxis label="Frequency, %" labelattrs=(size=**11**pt weight=bold family='Times New Roman')values=(**0** to **100** by **10**);

density Tl_ppb / type=kernel GROUP=preterm;

**run**;

**proc** **sgplot** data=data_graph NOAUTOLEGEND;

histogram V_ppb/ GROUP=preterm binstart=**42** binwidth=**3** transparency=**0.5**;

xaxis label="V, ppb" labelattrs=(size=**24**pt weight=bold family='Ariel');

yaxis label="Frequency, %" labelattrs=(size=**11**pt weight=bold family='Times New Roman')values=(**0** to **100** by **10**);

density V_ppb / type=kernel GROUP=preterm;

**run**;

**proc** **sgplot** data=data_graph NOAUTOLEGEND;

histogram U_ppb/ GROUP=preterm binstart=**42** binwidth=**3** transparency=**0.5**;

xaxis label="U, ppb" labelattrs=(size=**24**pt weight=bold family='Ariel');

yaxis label="Frequency, %" labelattrs=(size=**11**pt weight=bold family='Times New Roman')values=(**0** to **100** by **10**);

density U_ppb / type=kernel GROUP=preterm;

**run**;
